# Transcriptome analysis of an apple (*Malus* × *domestica*) yellow fruit somatic mutation identifies a gene network module highly associated with anthocyanin and epigenetic regulation

**DOI:** 10.1093/jxb/erv433

**Published:** 2015-09-28

**Authors:** Islam El-Sharkawy, Dong Liang, Kenong Xu

**Affiliations:** ^1^Horticulture Section, School of Integrative Plant Science, Cornell University, NYSAES, Geneva, NY 14456, USA; ^2^ Present address: Institute of Pomology & Olericulture, Sichuan Agricultural University, Chengdu, Sichuan 611130, China

**Keywords:** Apple, anthocyanin, *MdGST*, *MdMYB10*, methylation, somatic mutation, RNA-seq.

## Abstract

Using RNA-seq, this study analysed an apple (*Malus*×*domestica*) anthocyanin-deficient yellow-skin somatic mutant ‘Blondee’ (BLO) and its red-skin parent ‘Kidd’s D-8’ (KID), the original name of ‘Gala’, to understand the molecular mechanisms underlying the mutation. A total of 3299 differentially expressed genes (DEGs) were identified between BLO and KID at four developmental stages and/or between two adjacent stages within BLO and/or KID. A weighted gene co-expression network analysis (WGCNA) of the DEGs uncovered a network module of 34 genes highly correlated (*r*=0.95, *P*=9.0×10^–13^) with anthocyanin contents. Although 12 of the 34 genes in the WGCNA module were characterized and known of roles in anthocyanin, the remainder 22 appear to be novel. Examining the expression of ten representative genes in the module in 14 diverse apples revealed that at least eight were significantly correlated with anthocyanin variation. *MdMYB10* (MDP0000259614) and *MdGST* (MDP0000252292) were among the most suppressed module member genes in BLO despite being undistinguishable in their corresponding sequences between BLO and KID. Methylation assay of *MdMYB10* and *MdGST* in fruit skin revealed that two regions (MR3 and MR7) in the *MdMYB10* promoter exhibited remarkable differences between BLO and KID. In particular, methylation was high and progressively increased alongside fruit development in BLO while was correspondingly low and constant in KID. The methylation levels in both MR3 and MR7 were negatively correlated with anthocyanin content as well as the expression of *MdMYB10* and *MdGST*. Clearly, the collective repression of the 34 genes explains the loss-of-colour in BLO while the methylation in *MdMYB10* promoter is likely causal for the mutation.

## Highlight

An apple loss-of-colour fruit somatic mutation is likely the consequence of collective repression of a co-expression gene network module highly associated with anthocyanin and methylation in the promoter of *MdMYB10*.

## Introduction

Somatic mutations in woody crop species, known as ‘bud sports’ or simply ‘sports’, have been an important source of discovery of new cultivars or strains that are superior to the parent. In tree fruit, somatic mutations that alter overall reproductive growth characteristics, such as fruit-set behaviour, bearing habit, and fruit colour, size, shape and maturity, have been acknowledged previously (Petit and Hampe, 2006). Among them, fruit colour sport is the most frequently captured. For instance, two apple (*Malus* × *domestica*) cultivars, ‘Royal Gala’ and ‘Galaxy’, are bud sports of ‘Gala’ that were reported with fruit more coloured than the parent (Dickinson and White, 1986). Similarly, the light-red-skinned ‘Ruby Okuyama’ and the intense-rosy-skinned ‘Benitaka’ grapes were demonstrated to be bud sports of the white-skinned ‘Italia’ (Azuma *et al.*, 2009).

In plants, flavonoids are synthesized through a branched pathway yielding different subclasses of flavonoid compounds, such as anthocyanins, betalains, chlorophylls and carotenoids, each serving different functions in plant development, reproduction, defence, and protection against biotic/abiotic stresses (Macheix *et al.*, 1990; Koes *et al.*, 1994; Zhang *et al.*, 2013). In apple, anthocyanins, predominantly present in the cyanidin-3-galactoside form, are the major flavonoids induced during fruit maturation (Tsao *et al.*, 2003). From consumers’ perspectives, red skin apples provide not only essential cultivar differentiation, but also health benefit due to their antioxidant and other bioactive properties (Boyer and Liu, 2004).

Considerable effort has been invested in improving and clarifying the mechanisms underlying the red coloration of apple skin. Based on these efforts, three major associated factors have been proposed to be involved in anthocyanin accumulation. The first factor is the biosynthetic pathway, consisting of a number of enzymes that catalyse a sequential reaction for anthocyanin synthesis within the cytoplasm compartment. In apple, the expression of most genes involved in the anthocyanin biosynthetic pathway is positively correlated with anthocyanin accumulation (Honda *et al.*, 2002; Kim *et al.*, 2003; Xie *et al.*, 2014). However, such observation is not always the case in other higher plants. For example, in grapes and pears, only the action of the UDP-glucose:flavonoid 3-O-glucosyltransferase (UFGT) enzyme has been shown to be the key regulatory step in anthocyanin biosynthesis and the development of red coloration (Boss *et al.*, 1996; Kobayashi *et al.*, 2004; Wang *et al.*, 2013).

The second factor is the transcription regulation that coordinates the induction of anthocyanin biosynthesis during fruit development or in response to several environmental impacts such as light, temperature, hormones and stresses (Saure, 1990; Dong *et al.*, 1995; Lister and Lancaster, 1996; Venisse *et al.*, 2002; Whale and Singh, 2007; Manganaris *et al.*, 2008; Lin-Wang *et al.*, 2011; Qi *et al.*, 2011; Qian *et al.*, 2014). Anthocyanin biosynthesis is controlled by a transcription complex composed of two transcription factors that belong to the R2R3-MYB and the bHLH-MYC protein families, and a WD40 co-factor protein. The three proteins co-function by forming the MYB-bHLH-WD40 (MBW) complex to activate the expression of a downstream cascade of structural genes in the flavonoid/anthocyanin pathway (Gonzalez *et al.*, 2008; An *et al.*, 2012). Despite the critical contribution of bHLH and WD40 in the complex, the MYB protein is the key component in providing specificity for the subsets of anthocyanin biosynthetic genes and in determining the rate and distribution of red colour in apple (Zimmermann *et al.*, 2004; Espley *et al.*, 2009). Evaluation of several mutants provided strong evidence regarding the autonomous role of MYB (i.e. independent of the bHLH and WD40 partners) in mediating the transcription of genes involved in the early steps of the anthocyanin biosynthetic pathway (*CHS*, *CHI*, *F3H*, *F3’H* and *FLS*) that leads to the production of colourless dihydroflavonol compounds. However, the activation of genes functioning in the late steps of the pathway (*DFR*, *LDOX* and *UFGT*) that leads to the production of anthocyanins (colour pigmentation), requires the MBW complex (Baudry *et al.*, 2004; Stracke *et al.*, 2007; Gonzalez *et al.*, 2008; Petroni and Tonelli, 2011).

The third factor refers to the final step in the pathway, which is defined by anthocyanin transport from cytosol, where it is biosynthesized, into the vacuole, where it is stored. Two kinds of molecular actors are putatively associated with the vacuolar sequestration of anthocyanins: glutathione *S*-transferases (GSTs) and MATE-type transporters, named anthoMATEs (Winkel-Shirley, 2001; Koes *et al.*, 2005). Once anthocyanins are synthesized, they accumulate in the vacuole; and this localization is necessary for anthocyanins to function as pigments and to prevent them from oxidation (Marrs *et al.*, 1995).

Although significant progress has been made in revealing the anthocyanin biosynthesis and accumulation mechanisms, the fundamental understanding of the pathway regulation is still incomplete. One example is the nature of regulatory signals acting upstream of MBW, the direct regulators of those anthocyanin biosynthetic genes. It is not clear how the MBW complexes respond to different environmental factors, whether there are functional specificities for different MBW complexes, and what occurs in the crosstalk between different regulatory complexes. Also, enzymes involved in the synthesis of different anthocyanin molecules remain to be comprehensively elucidated. Characterizing plant mutants, including somatic mutations that exhibit phenotypic alterations in their pigmentation programme, should help better understand the mechanisms through which anthocyanins are regulated, biosynthesized and accumulated.

In this study, a rare anthocyanin-deficient yellow-skin apple mutant Blondee (BLO) and its red-coloured parent ‘Kidd’s D-8’ (KID), the original name of ‘Gala’, were characterized at four developmental stages. A series of RNA-seq and co-expression gene network analysis and methylation assays demonstrated that the loss-of-colour mutation in BLO is likely the consequence of collective repression of a gene network module of 34 genes that is highly associated with anthocyanin, and the methylation in *MdMYB10* promoter is likely the causal epigenetic factor for the mutation.

## Materials and methods

### Plant materials

Yellow apple ‘Blondee’ (BLO) is a patented somatic mutant or bud sport from ‘Kidd’s D-8’ (KID), the original name of apple cultivar ‘Gala’. BLO exhibits similar vegetative and reproductive growth as KID in terms of tree stature, flowering date, fruit development, ripening behaviour, and fruit size (Supplementary Fig. S1A). Mature KID fruit are often red with vertical strips. In contrast, BLO fruit look yellow with little or no red at maturity (Supplementary Fig. S1B). In order to determine the parent and sport relationship between KID and BLO, genetic fingerprinting was performed using 16 simple sequence repeat (SSR) markers (Supplementary Table S1) with three independently grafted trees each alongside ‘Fuji’ as a negative control.

Fruit samples of KID and BLO were collected in 2013 from 5-year-old trees grafted onto M.26 rootstock grown in the experimental orchard of Cornell University at Geneva, New York, USA. Briefly, 10−15 fruit from each of the two ‘Gala’ strains were sampled at four developmental stages, i.e. 16 July (S1), 7 August (S2), 26 August (S3), and 14 September (S4), respectively (Supplementary Table S2). Stage S4, which is defined by Cornell Starch Index 4−6 (Blanpied and Silsby, 1992), is equivalent to the fruit maturity stage for commercial harvest. The fruits were weighed and then the skin was carefully peeled and collected (samples from stage S1 contained more cortex tissues due to small fruit size). For 2014 fruit sampling, skin and flesh tissues were separated from at least 15 fruits of 14 *Malus* accessions, including KID and BLO at two developmental stages; immature (S2) and mature (S4). These accessions were selected according to the diversity in their skin and flesh colours (Supplementary Table S2). In different experiments, samples from 3−5 fruit per replicate with three independent biological replicates were collected. All samples were immediately frozen in liquid nitrogen and stored at −80°C for further analysis of both anthocyanin contents and gene expression profiles (by RNA-seq and qRT-PCR).

### Evaluation of anthocyanins

Lyophilized apple fruit samples, skin or flesh, were finely ground and ~100mg of ground tissues were homogenized in 10ml of methanol with 1% HCl. The homogenate was then kept for 24h at 4°C with shaking in dark conditions. All samples were centrifuged for 5min at 6,000 ×*g*. The methanol extracts were collected in a new tube, evaporated at 30°C under a stream of nitrogen, and the residue was immediately re-suspended in 1ml of 0.01N HCl. Then, the samples were filtered through a 0.2 µm polyethersulfone (PES) filter (Krackeler Scientific, Inc., Albany, NY, USA) and analysed using Agilent 1260 Infinity series HPLC (Agilent Technologies, Santa Clara, CA, USA) with a pentafluorophenyl (PFP) Kinetex® column (2.1×100mm, 100 Å, 2.6 µm particle size, Phenomenex Inc, Torrance, CA) as described previously (Manns and Mansfield, 2012). In brief, 5 µl of the filtered sample in 1:1 dilution was injected and run for 30min under 45ºC (column temperature) and 0.2ml/min (flow rate). Identification and quantification of anthocyanins were conducted using standards cyanidin (Cy)-3-galactoside, Cy-3-glucoside, Cy-3-arabinoside and Cy-3-rutinoside (Extrasynthese, Geney Cedex, France) (Supplementary Fig. S2). The data were obtained from three independent biological replicates.

### Nucleic acid extraction and RNA-seq library construction

Total RNA from the apple fruit skin or flesh tissues was extracted as described previously (Meisel *et al.*, 2005). All RNA extracts were treated with the RNase-Free DNase Set (Qiagen, Valencia, CA, USA) then cleaned up with the RNeasy Mini Kit (Qiagen). Genomic DNA (gDNA) was extracted from young leaves and different fruit tissues using the DNeasy Plant Mini Kit (Qiagen) or the method described for Citrus species (Cheng *et al.*, 2003). A total of 24 RNA-seq libraries (three biological replicates at four stages for both BLO and KID) were constructed as described previously (Bai *et al.*, 2014) using NEBNext Ultra Directional RNA Library Prep Kit for Illumina (New England Biolabs, Ipswich, MA). These libraries were multiplexed in an equal amount for single end 101-base sequencing in two lanes of HiSeq 2000 (Illumina, San Diego, CA) at the Cornell University Biotechnology Resource Center (Ithaca, NY).

### RNA-seq data analysis

Illumina sequencing of the pooled RNA-seq libraries yielded 24 FASTQ files of sequences (PRJNA287523/SRP062637) with a total of 327.5 million reads passed the Illumina Casava pipeline 1.8 (Supplementary Table S3). The reads were aligned to the rRNA reference sequences obtained from the SILVA rRNA database (Quast *et al.*, 2013) with the minimum sequence similarity of 0.95 and minimum length fraction of 1.0 to remove reads derived rRNA. The rRNA depleted reads of 307.9 million were then used for mapping against the improved apple reference transcriptome (Bai *et al.*, 2014) using CLC Genomics Workbench v7.5 (CLCBio, Cambridge, MA, USA) with the following parameters: the minimum similarity fraction of 0.98, the minimum length fraction of 0.8, and the maximum number of hits of 10. Gene expression levels were calculated and normalized by reads per kilobase of exon model per million mapped reads (RPKM) (Mortazavi *et al.*, 2008). Genes of RPKM>1.0 were defined to be expressed. For convenience, these novel transcripts in the improved reference transcriptome will be referred to ‘gene’ and named ‘G######’ as in ‘G101234’, and ‘MDP0000’ in the original gene IDs (e.g. MDP0000123456) will be abbreviated to ‘M’ (e.g. M123456) hereafter.

### Identification of differentially expressed genes and co-expression network modules

Differentially expressed genes (DEGs) were identified between BLO and KID at each of the four developmental stages as well as between immediately adjacent two stages within BLO and KID based on Baggerly’s test (Baggerly *et al.*, 2003) on RPKMs. Benjamini-Hochberg correction (Benjamini and Hochberg, 1995) was used to adjust the original P-values in Baggerly’s test to minimize the false discovery rate (FDR). A DEG is declared if the associated P_FDR_ < 0.05 was observed. For functional annotation of DEGs, the improved apple reference transcriptome (Bai *et al.*, 2014) was directly referred to, which was annotated with MapMan bins or functional classes (Thimm *et al.*, 2004). The highly co-expressed gene modules were inferred from the DEGs using weighted gene co-expression network analysis (WGCNA), an R package (Zhang and Horvath, 2005; Langfelder and Horvath, 2008). WGCNA network construction and module detection was conducted using an unsigned type of topological overlap matrix (TOM), a power β of 10, a minimal module size of 30, and a branch merge cut height of 0.25. The module eigengene (the first principal component of a given module) value was calculated and used to evaluate the association of modules with anthocyanin contents in the 24 samples. The most significant module (‘Pink’) of 34 genes with WGCNA edge weight >0.10 was represented using Cytoscape 3.1 (Saito *et al.*, 2012) and were also analysed using Network Analyzer (Assenov *et al.*, 2008).

### Sequence analysis of *MdMYB10* and *MdGST*

Full-length coding region cDNA and genomic DNA (including promoter) of *MdMYB10* and *MdGST* were isolated from KID and BLO using primers listed in Supplementary Table S1. PCR reactions were performed using Platinum Taq DNA Polymerase High Fidelity, following the manufacturer’s instructions (Invitrogen, Carlsbad, CA, USA). The PCR products were then purified using the MinElute Reaction Cleanup Kit (Qiagen). DNA fragments from three independent replicates were cloned using the pGEM-T easy vector (Promega, Madison, WI, USA), and sequenced using ABI 3730xl (Applied Biosystems, Foster City, CA, USA). Promoter sequence analysis was performed using the PLACE Signal Scan Search online database (Higo *et al.*, 1999).

### Methylation assay

An McrBC-PCR approach was used to analyse the methylation levels of in the *MdMYB10* and *MdGST* promoter regions. One microgram (µg) of genomic DNA (*g*DNA) isolated from KID and BLO fruit skin samples collected in 2013 and 2014 were digested with methylation-specific endonuclease enzyme McrBC (New England Biolabs), according to the manufacturer’s instructions, with three biological replicates. For the negative control, Guanosine-5′-triphosphate (GTP) was replaced by water in the reaction. The digested *g*DNA was used as template for semi-quantitative PCR analysis. The *MdMYB10* and *MdGST* promoter sequences were divided into five and seven fragments, respectively; and amplified with their respective primers (Supplementary Table S1). The amount of amplicons was visualized by agarose gel (1.5%) electrophoresis and used to estimate the methylation levels of the corresponding regions.

Bisulfite sequencing analysis was performed as described by Telias *et al.* (2011) with three biological replicates. Briefly, 750ng of *g*DNA from KID and BLO fruit skin samples were treated using the EZ DNA Methylation-Gold Kit (Zymo Research, Irvine, CA, USA). Using the treated *g*DNA as template, the targeted *MdMYB10* promoter fragments were amplified using Platinum Taq DNA Polymerase High Fidelity (Invitrogen) with degenerate primers (Supplementary Table S1), ligated into pGEM-T Easy vector (Promega), and then sequenced as described above. Sequences of nine independent clones per fragment obtained from three independent PCR reactions were analysed using the online software Kismeth (Gruntman *et al.*, 2008), and the methylation level of the targeted fragments was calculated based on the percentage of detected cytosines in methylated DNA relative to control. To determine whether the methylation levels are dependent on fruit development stages, bisulfite sequencing analysis was also performed using *g*DNA from the 2013 fruit skin samples of KID and BLO at the four stages. Three independent PCR reactions from three different biological replicates were purified and sequenced for analysis.

### Quantitative reverse transcription RT-PCR analysis

DNase-treated RNA (2 µg) was reverse transcribed in a reaction of 20 µl using High Capacity cDNA Reverse Transcription Kit (Applied Biosystems, Foster City, CA, USA). Gene-specific primers were designed using Primer Express (v3.0, Applied Biosystems) (Supplementary Table S1). Quantitative reverse transcription PCR (qRT-PCR) assays were performed using 20ng of cDNA and 300nM of each primer in a 10 µl reaction with SYBR Green I Master Mix (Roche, Indianapolis, IN, USA). Three biological and three technical replicates for each reaction were analysed on a LightCycler 480 instrument (Roche) with a first step of 95°C for 5min followed by 40 cycles of 95°C for 10 s, 60°C for 10 s, and 72°C for 20 s. Melting curves were generated using the following programme: 95°C for 15 s, 60°C for 15 s, and 95°C for 15 s. Transcript abundance was quantified using standard curves for both target and reference genes, which were generated from serial dilutions of PCR products from corresponding cDNAs. Transcript abundance was normalized to the reference gene *MdActin* that showed high stability across the different apple genotypes and tissues used in this study.

### Statistical analyses

Statistical analyses were performed using JMP Pro10 (SAS, Cary, NC, USA). For correlation analysis, Pearson correlation coefficient (*r*) was calculated and a two-tailed test was carried out. For hierarchical cluster analysis, Ward’s method was used (Milligan, 1980).

### Genetic mapping of yellow fruit

Genetic mapping of yellow fruit was conducted at maturity stage in 2013 in population ‘GMAL 4595’ derived from cross ‘Royal Gala’×PI 613988 (*M. sieversii*). The seed parent ‘Royal Gala’ is also a sport mutation from KID. Mapping was conducted using the Kruskal-Wallis method available in MapQTL (Van Ooijen *et al.*, 2002). Yellow fruit was scored with value ‘1’ and coloured (non-yellow) fruit with value ‘2’. The parental genetic maps were constructed previously (Wang *et al.*, 2012) of 287 SSR markers in total.

## Results

### Genetic fingerprinting of KID and BLO

To ascertain the parent and sport relationship between KID and BLO, 16 randomly selected SSR markers were tested. The genetic fingerprinting data did not show any differences between KID and BLO, while a clear difference between Fuji and KID/BLO was observed in 11 of the 16 markers (Supplementary Fig. S3A), confirming the parent and sport relationship. Fruit size was also obtained to evaluate the relationship. The fruit sampled from KID were larger (0.05>*P*>0.01) than those from BLO at S1, S2, and S3; however, no significant difference (*P*=0.646) at maturity (S4) was observed (Supplementary Fig. S3B). At maturity (S4), Cornell Starch Index of BLO was 4.7, slightly more advanced than that of KID (3.8), but this difference was insignificant (*P*=0.218) (Supplementary Fig. S3C).

### Quantitation of anthocyanin contents in the two ‘Gala’ strains

**Fig. 1.**
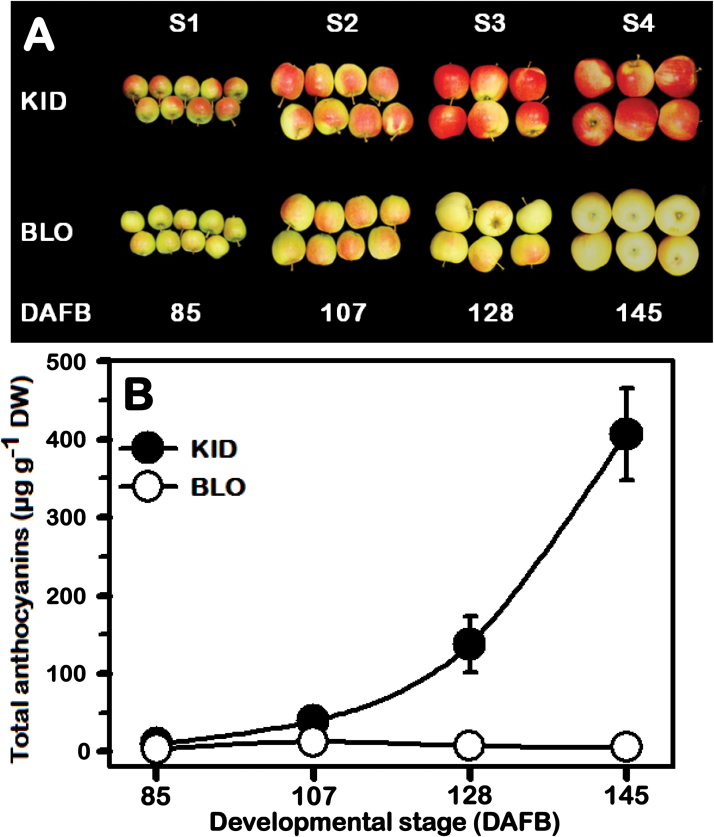
(A) Close-up views of KID and BLO fruit at four developmental stages (S1–S4) used for anthocyanin quantification, transcriptome profiling and methylation assays. (B) The changes in anthocyanin accumulation in the fruit skin of KID and BLO during development and maturation. Each value represents a mean ±SD of three independent biological replicates. This figure is available in colour at *JXB* online.

Visual inspection of fruit skin colour during development revealed that both ‘Gala’ strains slightly turned red at S1, with visibly more intense red colour in KID (Fig. 1A). In KID, the red coloration was increased at S2, a pattern that progressively advanced at much higher levels through subsequent stages (S3 and S4), resulting in red coloured fruit at maturity. BLO fruit behaved similarly to that of KID until the S2 stage, but with markedly lower red colour intensity. Later, the fruit displayed a significant decline in pigmentation, resulting in colour change from light red to yellow at maturity.

Consistent with the visual inspection, analysis of fruit skin anthocyanin contents by HPLC demonstrated distinctive anthocyanin accumulation profiles between the two strains, particularly in terms of concentration. Among several types of anthocyanin detected through the different developmental stages, the cyanidin 3-galactoside form was the predominant contributor to the skin colour of KID (92.5%) and BLO (100%). Other types of anthocyanins were detected, but only in mature KID fruit skin at S3 and S4 (Supplementary Table S4). In both ‘Gala’ strains, the levels of total anthocyanin were initially low and steadily increased during early developmental stages (S1 to S2). A clear divergence in anthocyanin accumulation behaviour between the two strains occurred thereafter (Fig. 1B; Supplementary Table S4). In KID fruit, anthocyanin content sharply increased along with maturation progression. In contrast, anthocyanins gradually reduced during BLO fruit maturation, resulting in significantly lower levels at S3 and S4. Relative to BLO, the total anthocyanin levels were ~3-fold higher in KID fruit skin during the S1 and S2 stages; however, during S3 and S4 it reached ~20- and ~77-fold higher levels, respectively (Fig. 1B; Supplementary Table S4).

### Characterization of the BLO and KID transcriptomes

Mapping the rRNA depleted 307.9 million RNA-seq reads from the 24 samples of BLO and KID against the improved apple reference transcriptome (Bai *et al.* 2014) showed that 222.3 million reads (72.2%) were mapped in total and 185.5 million (60.3%) were mapped uniquely (Supplementary Table S3). The mean mapped reads per sample were 9.3±1.5 million in total and 7.7±1.2 million in unique. Overall, the mapped reads enabled identification of a total of 32,019 genes expressed with RPKM>1.0 at least in one of the four stages between BLO and KID (Supplementary Fig. S4). The total number of expressed genes of BLO was 31 263 with 28 873 at S1, 26 562 at S2, 25 950 at S3, and 23 573 at S4 (Supplementary Fig. S4A). These numbers were similar in KID, i.e. the total number of expressed genes was 30 697 (566 genes or 1.8% less than that of BLO) with 28 316 at S1, 26 606 at S2, 25 516 at S3, and 23 357 at S4 (Supplementary Fig. S4B). These observations suggested that the number of expressed genes considerably decreased during fruit development and maturation in both ‘Gala’ strains. Despite the similarities in the numbers of expressed genes between BLO and KID, there were many genes expressed uniquely to each strain at each of the four stages (Supplementary Fig. S4C, D). In addition, a large number of genes expressed uniquely were also observed between two adjacent stages within BLO or KID (Supplementary Fig. S4A, B).

### Identification of differentially expressed genes between BLO and KID

**Fig. 2.**
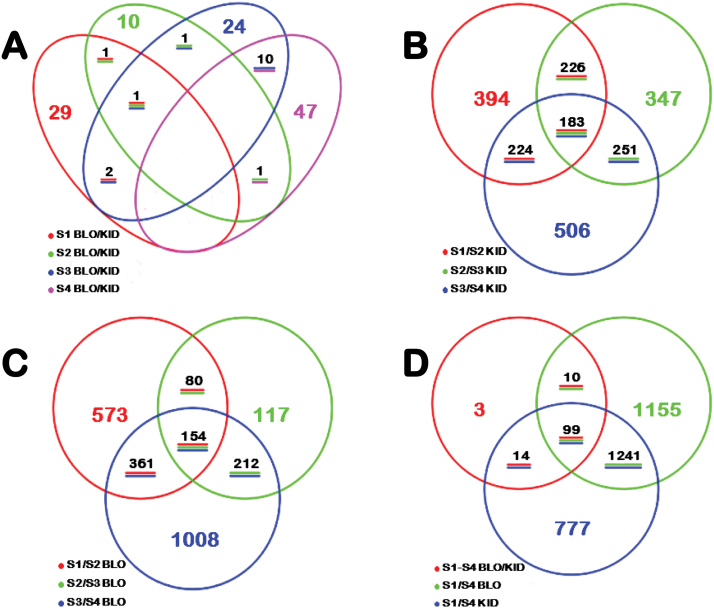
Venn diagram representation of the number of differentially expressed genes (DEGs) identified by RNA-seq analysis in the skin tissues of BLO and KID fruit. (A) Number of DEGs between BLO and KID at each of the four developmental stage (S1–S4). (B) Number of DEGs between two adjacent stages in KID. (C) Number of DEGs between two adjacent stages in BLO. (D) Combined number of DEGs from panels A, B and C. This figure is available in colour at *JXB* online.

Four pairwise transcriptome comparisons were made at each of the four stages to identify DEGs between BLO and KID. This allowed identification of 143 (126 non-redundant) DEGs (*P*_FDR_<0.05), which included 15 genes deferentially expressed at two stages and one in three stages (Fig. 2A). There were 47 DEGs in the two early stages, with 33 at S1 and 14 at S2, less than one half of the DEGs (96) observed at the two late stages (38 at S3 and 58 at S4).

Since the anthocyanin contents were progressively increased in KID from S1 through S4, it would be important to also identify DEGs between adjacent stages (i.e. S1 vs. S2, S2 vs. S3, and S3 vs. S4) within BLO and KID. In BLO, this analysis yielded 3466 DEGs (2,505 non-redolent), including 1168 between S1 and S2, 563 between S2 and S3, and 1735 between S3 and S4 (Fig. 2B). In KID, it uncovered 3198 DEGs (2131 non-redolent), including 1027 between S1 and S2, 1007 between S2 and S3, and 1164 between S3 and S4 (Fig. 2C). Overall, 3299 non-redundant DEGs were identified (Fig. 2D). Remarkably, 99 of 126 non-redundant DEGs identified between BLO and KID were also among the DEGs identified between the adjacent stages within both BLO and KID (Fig. 2D).

### Identification of WGCNA modules associated with anthocyanin contents

**Fig. 3.**
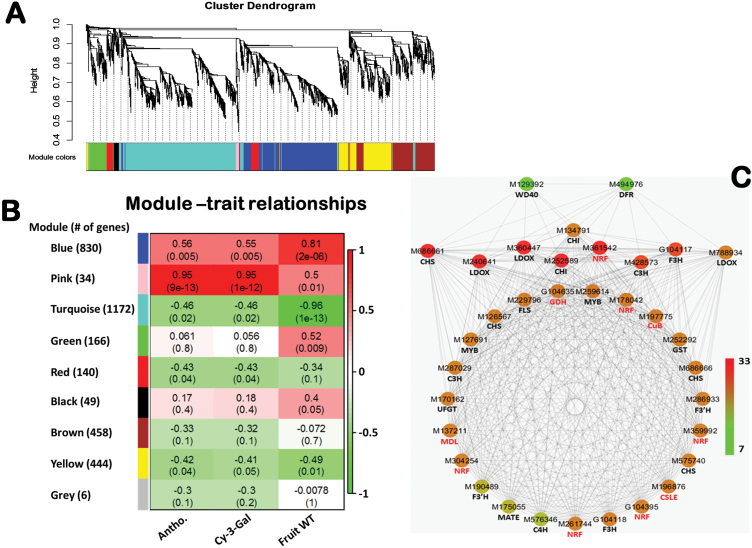
Weighted gene co-expression network analysis (WGCNA) of differentially expressed genes (DEGs) identified from the two ‘Gala’ strains over four developmental stages. (A) Hierarchical cluster tree showing nine modules of co-expressed genes. Each of the 3299 DEGs is represented by a leaf in the tree, and each of the nine modules by a major tree branch. The lower panel shows modules in designated colours, such as ‘Blue’, ‘Pink’, ‘Turquoise’ and others. Note that module ‘Grey’ is for unassigned genes. (B) Module-anthocyanin/fruit weight correlations and corresponding *P*-values (in parenthesis). The left panel shows the nine modules and the number of module member genes. The colour scale on right shows module-trait correlation from –1 (green) to 1 (red). (C) Cytoscape representation of co-expressed genes with edge weight ≥0.10 in module ‘Pink’. The edge number of the genes ranges from 7 to 33 (colour-coded by the scale on right from green through red). Member gene IDs and common names are given. Note that genes of IDs with leading letter ‘G’ are those referred to novel transcripts in the improved apple reference transcriptome (Bai *et al.*, 2014), and the leading letters and numbers ‘MDP0000’ in existing apple gene IDs are abbreviated as ‘M’, e.g. M123456 is an abbreviated ID for MDP0000123456. Gene name with ‘NRF’ refers to genes with non-reported function.

A weighted gene co-expression network analysis (WGCNA) was performed with the 3299 non-redundant DEGs identified above, leading to identification of nine WGCNA modules (Fig. 3A, B). Analysis of the module-trait relationships revealed that module ‘Pink’ of 34 genes was highly correlated with both total anthocyanin (*r*=0.95, *P*=9.0×10^–13^) and Cy-3-galactoside (*r*=0.95, *P*=1×10^–12^) contents while moderately correlated with fruit weight (*r*=0.50, *P*=0.01) in the 24 samples (Fig. 3B). Therefore, the 34 genes of module ‘Pink’ were considered to have an important role in anthocyanin accumulation in apple skin, and their collective repression would lead to loss-of-colour in BLO. Cytoscape representation of the 34 genes with WGCNA edge weight >0.10 indicated that these genes were highly connected, as 32 of the 34 genes had 28 or more edges and only two [*M129392* (i.e. MDP0000129392)*-MdWD40* and *M494976-MdDFR*] had low edge number (7 and 10, respectively) (Fig. 3C). Modules ‘Blue’ of 830 genes and ‘Turquoise’ of 1172 genes appeared to be highly associated with fruit weight although the trait is not directly related to the objectives of this study. The other six modules did not appear to be associated with anthocyanin accumulation and/or fruit weight.

### Functional annotation of the WGCNA module highly associated with anthocyanin

**Fig. 4.**
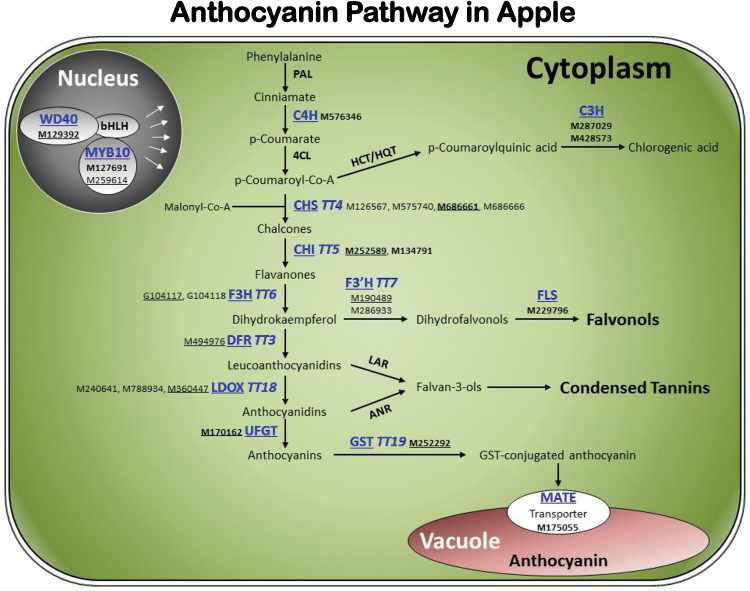
Diagram of the flavonoid/anthocyanin pathway assigned with 24 genes from the WGCNA module ‘Pink’. The proteins with names shown in blue and underlined are encoded by the 24 genes, including 12 previously characterized (in regular font) and 12 newly identified in this study (in bold font). Genes with IDs underlined were chosen for qRT-PCR assays. Please refer to the abbreviation section for the full names of proteins or genes abbreviated in the figure. This figure is available in colour at *JXB* online.

**Fig. 5.**
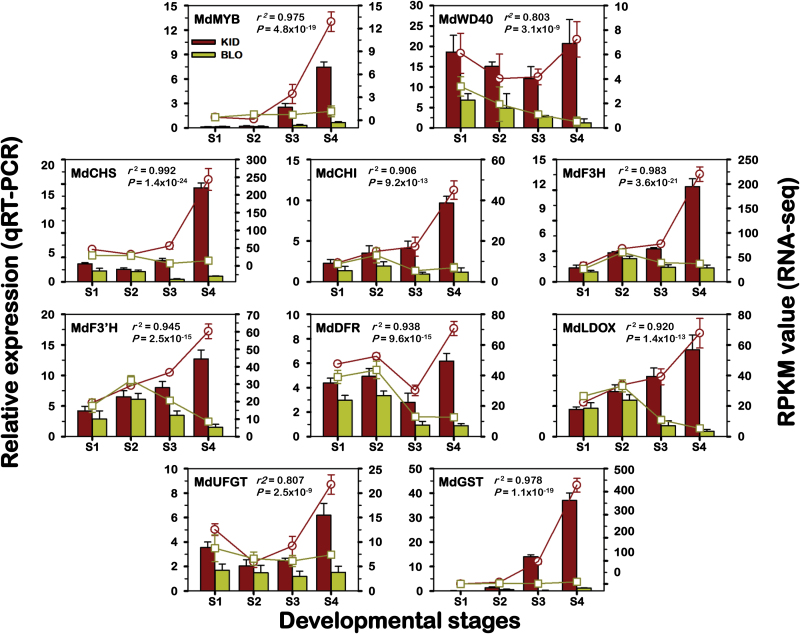
Comparison of expression profiles of ten representative genes from module ‘Pink’ as measured by RNA-seq and qRT-PCR. The ten genes are assigned to the flavonoid/anthocyanin pathway in Fig. 4, including five previously characterized and five uncharacterized genes. Columns represent expression determined by qRT-PCR (left *y*-axis), while lines represent expression by RNA-seq in RPKM values (right *y*-axis). The *x*-axis in each chart represents the four developmental stages (S1–S4). For qRT-PCR assay, the mean was calculated from three biological replicates each with three technical replicates (*n*=9). Standard curves were used to calculate the number of target gene molecules per sample. These were then normalized relative to the expression of *MdAct*. For RNA-seq, each point is the mean of three biological replicates. Correlations between qRT-PCR and RNA-seq expressions were calculated and their associated *P*-values are indicated. Error bars show SD. This figure is available in colour at *JXB* online.

Based on MapMan function annotation, the 34 genes in module ‘Pink’ could be classified into three main groups (Table 1). Group 1 comprises four genes (11.8%), which encode a cellulose synthase (CSLE), a glutamate dehydrogenase (GDH), a copper ion binding (CuB) protein, and a mandelonitrile lyase (MDL), respectively. Genes encoding such proteins have not been reported to contribute to the anthocyanin pathway. Group 2 covers six genes (17.6%) with unknown functions. Group 3 includes the remaining 24 genes (70.6%) encoding proteins putatively associated with anthocyanin regulation, biosynthesis and transport (Table 1; Fig. 4). Comparison of the 24 anthocyanin-related genes with previously reported homologues identified 12 genes that have not been characterized so far (Supplementary Table S5). Examining their WGCNA gene significance (GS) for anthocyanin (i.e. correlation with the trait) showed that the 24 genes had a range of GS with *MdMYB10* (0.989) and *MdGST* (0.988) being the highest and *MdWD40* (0.588) and *MdDFR* (0.622) the lowest (Table 1). Analysis of ten of the 34 genes using qRT-PCR demonstrated that the relative expression and the RPKMs for each gene were significantly correlated (*r* ^2^>0.803, *P*<3.1×10^–9^), confirming the expression measured by RNA-seq (Fig. 5). The ten genes included five that were originally identified in this study while the other five were previously reported (Supplementary Table S5).

**Table 1.** List of member genes in the WGCNA module ‘Pink’ highly associated with anthocyanin contents The gene ID in bold font represents the genes selected for further qRT-PCR analysis.

**Gene ID**	**Gene name**	**Function**	**Fold change (RPKM**)				**Gene significance**	
			**S1**	**S2**	**S3**	**S4**	**Anthocyanin**	***P***
M196876	MdCSLE	Cellulose synthase-like family E	0.89	1.03	1.39	1.90	0.8791	1.57E-08
G104635	MdGDH	Glutamate dehydrogenase	1.19	1.06	1.05	2.38	0.9264	8.44E-11
M197775	MdCuB	Copper (Cu) ion binding	0.99	1.10	0.94	1.87	0.7584	1.75E-05
M137211	MdMDL	Flavoprotein (*R*) (+)-mandelonitrile lyase	0.29	3.08	0.25	4.52	0.9014	1.87E-09
M178042	-	Unknown	1.00	1.06	1.10	1.63	0.8894	6.19E-09
M261744^a^	-	Unknown	-	-	-	-	0.9476	2.22E-12
M361542	-	Unknown	2.47	1.39	4.17	4.86	0.8887	6.61E-09
M359992	-	Unknown	2.03	14.77	4.17	2.29	0.8705	3.21E-08
M304254	-	Unknown	3.76	1.49	0.75	6.73	0.8524	1.24E-07
G104395	-	Unknown	1.14	0.99	4.71	21.07	0.9903	2.38E-20
**M259614** ^**b**^	MdMYB10	R2R3-MYB transcription factor	0.70	1.85	19.37	23.82	0.9885	1.47E-19
M127691^b^			1.02	0.19	4.63	11.01	0.9537	5.84E-13
**M129392**	MdWD40	TRANSPARENT TESTA GLABRA1	1.80	2.07	3.81	15.89	0.5878	2.52E-03
M576346	MdC4H	Cinnamate 4-hydroxylase	1.51	1.05	1.14	1.38	0.7757	8.44E-06
M287029	MdC3H	4-Coumarate 3-hydroxylase	1.18	0.84	0.66	7.61	0.8917	4.97E-09
M428573			1.11	1.10	1.09	8.05	0.9122	5.44E-10
M126567	MdCHS	Chalcone synthase	1.44	1.04	2.00	4.06	0.8896	6.09E-09
M575740			1.36	1.05	1.82	2.13	0.8059	2.01E-06
**M686661**			1.58	1.13	8.07	16.64	0.9455	3.35E-12
M686666			1.45	1.14	2.82	4.72	0.9072	9.86E-10
M134791	MdCHI	Chalcone isomerase	1.02	1.15	1.81	4.00	0.7294	5.26E-05
**M252589**			1.06	1.15	3.16	6.49	0.9515	9.67E-13
**G104117**	MdF3H	Flavone 3-hydroxylase	1.25	1.13	1.97	5.91	0.9607	1.00E-13
G104118			1.34	0.82	1.86	4.23	0.9059	1.14E-09
**M190489**	MdF3′H	Flavonoid 3′-hydroxylase	1.10	0.90	1.78	7.03	0.8920	4.85E-09
M286933			1.30	0.86	1.44	2.17	0.8148	1.25E-06
M229796	MdFLS	Flavonol synthase	1.63	2.12	3.34	5.31	0.9217	1.62E-10
**M494976**	MdDFR	Dihydroflavonol reductase	1.22	1.20	2.32	5.58	0.6215	1.19E-03
M240641	MdLDOX	Leucoanthocyanidin dioxygenase	1.15	1.34	7.79	24.55	0.9316	3.86E-11
**M360447**			0.89	1.34	13.48	32.16	0.9511	1.04E-12
M788934			0.84	1.02	3.58	12.72	0.8333	4.32E-07
**M170162**	MdUFGT	UDP-glucosyl transferase	1.44	0.89	1.50	2.94	0.8316	4.78E-07
**M252292**	MdGST	Glutathione *S*-transferase	1.30	2.45	53.59	46.31	0.9884	1.65E-19
M175055	MdMATE	Multidrug and toxic compound extrusion transporters	1.99	1.42	1.44	2.02	0.8599	7.24E-08

^a^ The fold change could not be determined due to transcription absence from BLO strain.

^b^ M127691 is almost a truncated version of M259614 based on their sequences, and the two genes are physically separated by less than 5kb on chromosome 9 in the apple genome, indicating they are likely allelic genes.

### Genetic and epigenetic characterization of MdMYB10 and MdGST

To shed light on how the WGCNA module ‘Pink’ may operate in controlling anthocyanin accumulation in the two ‘Gala’ strains, *MdMYB10* (M259614, i.e. MDP0000259614) and *MdGST* (M252292, i.e. MDP0000252292) were characterized in more detail. These two genes were not only among the few of the highest GS for anthocyanin in the module ‘Pink’, but also among those of the largest RPKM fold-changes (>23-fold in *MdMYB10*; >46-fold in *MdGST*, the largest) between the two stains at maturity (S4) (Table 1; Fig. 5).

First, the cDNA coding sequences as well as the genomic DNA sequences of the two genes were isolated from BLO and KID. The *g*DNA included a promoter region of 2585 and 2725 bases upstream from the translation initiation site of *MdMYB10* and *MdGST*, respectively. Sequence analysis indicated that there were no base mutations in the cDNA or gDNA sequences between the two strains except for a few random sequence errors, suggesting that the genetic variation was not the cause of suppression of *MdMYB10* and/or *MdGST* mRNAs in BLO.

Next, methylation levels in the promoter region of the two genes were investigated. Genomic DNA samples prepared from the KID and BLO skin tissues from two growth seasons (2013 and 2014) were digested with endonuclease McrBC. The McrBC treated and mock-digested *g*DNA templates were compared using a semi-quantitative PCR approach. For *MdGST*, five fragments that cover the whole promoter region (from −2725 to +116) were assessed. The results demonstrated no visible differences in amplicon levels, suggesting that KID and BLO had similar methylation levels in their promoter region of *MdGST* (Supplementary Fig. S5), indicating epigenetic factors were also unlikely responsible for the repression of the *MdGST* transcripts in BLO. However, further analysis of the promoter sequence of *MdGST* identified 19 MYB- and 11 MYC-binding sites (Supplementary Table S6), signifying that the expression of *MdGST* might be regulated by MYB and/or bHLH (MYC) transcription factors.

**Fig. 6.**
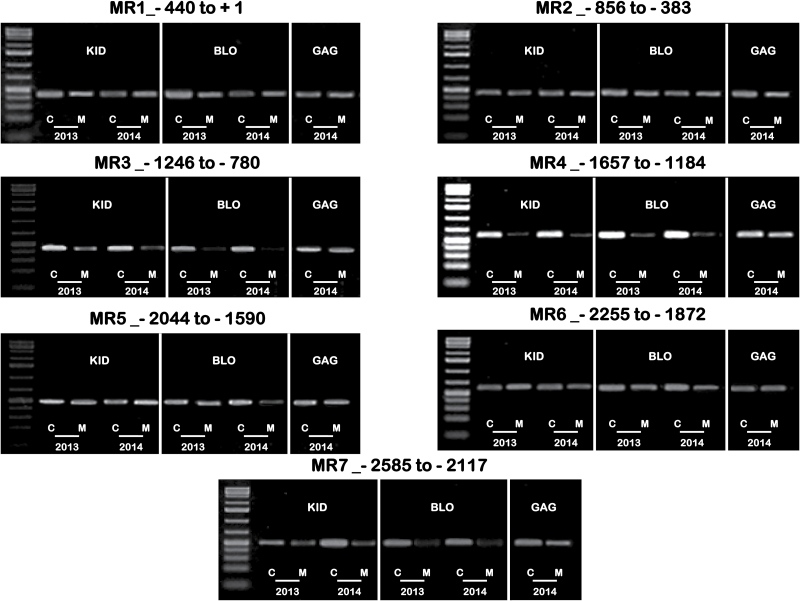
Identification of regions in the promoter of *MdMYB10* with different methylation levels between KID and BLO using the McrBC-PCR approach. The *g*DNA from the skin of mature fruit (S4) collected in 2013 and 2014 was used. The promoter was divided into seven regions (MR1–MR7), and the location of each region is indicated. The number of cycles necessary for exponential, but non-saturated, PCR amplification was determined for each region using the negative control template (GTP replaced by water). Letters M and C indicate the McrBC digestion reactions with or without GTP (i.e. negative control), respectively. Reactions were performed in three independent triplicate PCR reactions using templates derived from three independent McrBC digestions.

For methylation assay of *MdMYB10* (Fig. 6), another ‘Gala’ bud sport named ‘GaleGala’ that exhibits more skin red-coloration than KID was included in the 2014 season. A total of seven fragments that comprise the whole promoter region (from −2585 to +1) were evaluated. The initial results using the McrBC approach indicated that the regions, designated MR3 (−1246 to −780) and MR7 (−2585 to −2117), were clearly different in methylation activity among the three ‘Gala’ strains. Another region, designated MR5 (−2044 to −1590), showed visible variation in methylation of BLO, but only during the 2014 growing season and only in one of the three replicates. Further, the region MR4 (−1657 to −1184) exhibited high methylation activity and resulted in almost complete digestion of the amplified DNA, but only in BLO and KID strains (not in ‘GaleGala’). However, all other tested regions showed similar low methylation levels (Fig. 6).

**Fig. 7.**
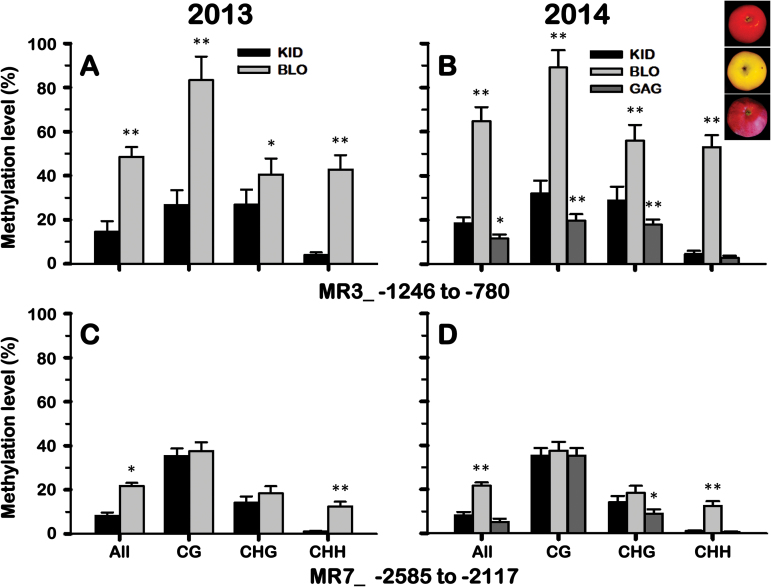
Bisulfite sequencing analysis of cytosine methylation levels of *MdMYB10* promoter in the skin samples of KID and BLO fruit collected in 2013 and 2014. ‘GaleGala’ (GAG) fruit skin samples were collected only in 2014. A close-up view of KID, BLO and GAG fruit is arranged in order from top to bottom, respectively. The charts show the methylation levels of different cytosine contexts in the MR3 (A and B) and MR7 (C and D) regions. Each data point represents a mean ±SD of three independent *g*DNA extractions with three independent technical replicates. Nine independent clones from each reaction were sequenced and analyzed. In the *x*-axis, ‘All’ refers to overall methylated cytosines, while CG, CHG and CHH refer to the three different contexts of cytosines, in which H represents nucleotide A, C or T. Statistical differences were assessed using Student’s *t*-test. Asterisks ‘*’ and ‘**’ indicate significance levels at *P*<0.05 and *P*<0.01, respectively. This figure is available in colour at *JXB* online.

To validate these results, the methylation levels of *MdMYB10* promoter from the fruit skin of KID, BLO and ‘GaleGala’ were further analysed using bisulfite sequencing. However, only the regions of MR3, MR5 and MR7, which showed visible differences in methylation levels between KID and BLO strains, were tested. Analysis of sequence data indicated that MR5 region displayed very low methylation activity during the two growing seasons; so it was considered that the occasional variations detected by McrBC were likely due to artifacts. The MR3 region showed diverse methylation levels among the three ‘Gala’ strains (Fig. 7A, B). The overall methylation level of the MR3 region was 70% lower in the fruit skin of KID than in that of BLO. Moreover, the methylation of CG, CHG, and CHH cytosines (where H is A, C or T) were 68%, 34%, and 91% lower in KID than in BLO, respectively. Consistent with the previous results, the highly coloured ‘GaleGala’ sport displayed a 38% lower number of methylated cytosines than KID, and this was mainly due to reduced methylation of CG (49% less) and CHG (45% less) contexts in ‘GaleGala’. However, the methylation of CHH cytosine did not show a significant difference between KID and ‘GaleGala’ (Fig. 7B).

The MR7 region showed much reduced methylation activity compared with the MR3 region (Fig. 7C, D). However, the methylation level in the MR7 region was 47% lower in the fruit skin of KID than that of BLO. This was mainly due to a 92% decrease in CHH cytosine methylation in KID as methylation of CG and CHG cytosines was not significantly different between BLO and KID. The MR7 region exhibited similar methylation activity between KID and ‘GaleGala’ although ‘GaleGala’ was 37% lower in CHG cytosine methylation than KID (Fig. 7D).

Analysis of overall methylated cytosines in MR3 and MR7 indicated that methylation could be detected in all the three cytosine contexts although CHH (47.5%) represented the most discriminatory context that can distinguish BLO from KID, followed by CG (12.3%), and CHG (6.8%). Between the two growing seasons, the skin of KID and BLO fruit exhibited similar methylation patterns although the methylation levels were significantly higher in 2014, but this made the differences in methylation activity between BLO and KID greater.

To determine whether the observed differences in *MdMYB10* promoter methylation levels are associated with the alterations in *MdMYB10* and *MdGST* transcription as well as anthocyanin accumulation, a correlation analysis was performed using the methylation levels in the MR3 and MR7 regions, respectively (Supplementary Table S7). The methylation of the MR3 and MR7 regions each displayed significant negative correlation with either anthocyanin accumulation (*r*MR3=−0.8002, *P*=1.34×10^–5^; *r*MR7=−0.8612, *P*=3.73×10^–5^) or the mRNA levels of *MdMYB10* (*r*MR3=−0.7638, *P*= 5.59×10^–5^; *r*MR7=−0.8492, *P*=6.19×10^–5^) and *MdGST* (*r*MR3=–0.7755, *P*=3.63×10^–5^; *r*MR7=–0.7196, *P*=2.50×10^–3^) during stages S3 and S4. Since there are at least 19 MYB-binding sites in the *MdGST* promoter region, such significant correlations strongly suggest that *MdMYB10* may regulate the expression of *MdGST*, and the dramatic suppression of *MdGST* in BLO is likely caused by low expression of *MdMYB10* due to high methylation in the MR3 and MR7 regions.

### Methylation of the promoter of *MdMYB10* in developing fruit

**Fig. 8.**
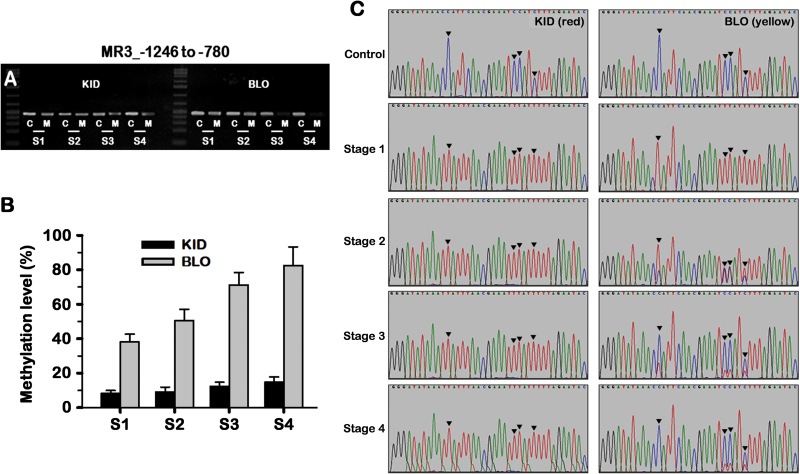
Methylation in the promoter MR3 region of *MdMYB10* at four developmental stages (S1–S4) in fruit skin of KID and BLO. (A) Methylation levels as detected by McrBC-PCR. The experiment was conducted as described in Fig. 6. Letters M and C indicate the McrBC digestion reactions with or without GTP (negative control), respectively. (B) Overall methylation levels as detected by bisulfite sequencing. The experiment was conducted as described in Fig. 7. (C) Sections of bisulfite sequencing traces. The arrows indicate the positions where the methylation occurred in cytosines, resulting in changes in nucleotide composition.

To examine whether there is alteration in the dynamics of methylation in the *MdMYB10* promoter during fruit development and maturation, the methylation activity of the MR3 region was assessed in the fruit skin of BLO and KID over the four stages using the McrBC approach. Data analysis indicated that BLO and KID showed constant low methylation levels during early developmental stages S1 and S2. A visible increase in methylation activity was detected in both BLO and KID at S3, but with much higher levels in BLO, causing almost complete digestion of the amplified template DNA (Fig. 8A).

Further, the bisulfite sequencing approach demonstrated that the fruit skin of BLO and KID exhibited markedly different methylation levels and behaviour during fruit development. The skin of KID fruit displayed very low and almost constant methylation activity during fruit development and maturation, since the majority of cytosines that were converted into thymines at S1 remained thymines throughout the subsequent developmental stages. The overall methylation levels in the KID fruit skin were estimated at 8.3%, 9.1%, 12.3% and 14.7% at the four developmental stages, respectively (Fig. 8B). However, in the BLO fruit skin, the corresponding methylation levels were 38.1%, 50.5%, 71.1%, and 82.5%, respectively, suggesting a much higher methylation level at S1 and more dramatic increases of methylation activity thereafter and through maturation. From S2, visible alterations in nucleotide composition occurred through detection of minor cytosine peaks overlapping with those of thymine (Fig. 8C). The height of cytosine peaks progressively increased along with fruit maturation, indicating a gradually increased methylation activity, resulting in more cytosine detected at S4. In contrast, this pattern of methylation was rarely detectable during KID fruit development. These data suggested that the methylation of the *MdMYB10* promoter is developmentally regulated.

### Expression profile of the ten selected genes in diverse apples of varying fruit colour phenotypes

**Fig. 9.**
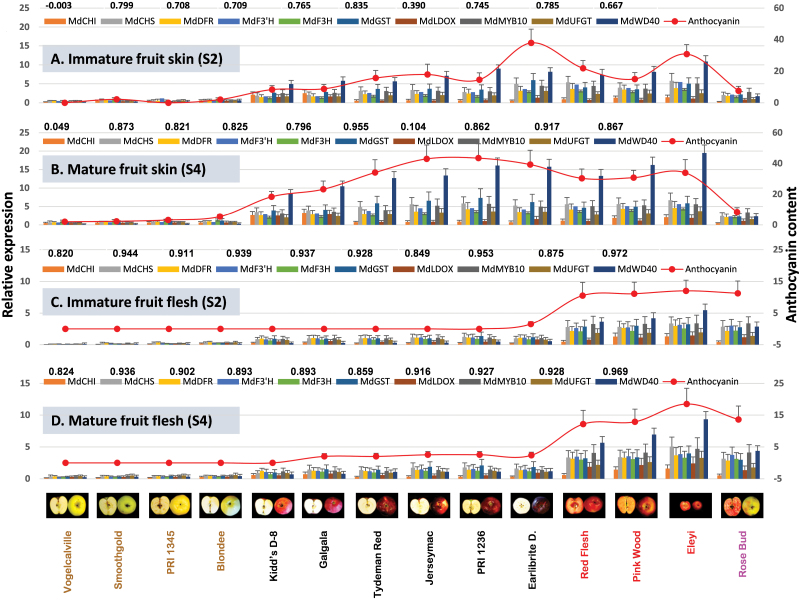
Relationships between anthocyanin contents and transcript levels of the ten representative genes from module ‘Pink’ in 14 *Malus* accessions of varying colours in fruit skin and flesh. The ten genes are the same as those used in Fig. 5, which are listed in Table 1 as well as in Fig. 4. For each accession, the expression was determined in two developmental stages immature (S2) and mature (S4) of skin (A and B) and flesh (C and D) tissues. Details of qRT-PCR analysis are as described in Fig. 5. Anthocyanin levels are indicated by red lines. The *x*-axis in each chart is the same and represents the 14 *Malus* accessions as indicated by their fruit close-up views and names at the bottom panel, which are arranged in four groups (distinguished by colour): 1, yellow skin/white flesh; 2, red skin/white flesh; 3, red skin/red flesh; and 4, yellow skin/red flesh. The left *y*-axis represents relative expression levels determined by qRT-PCR, and the right *y*-axis represents anthocyanin content (µg g^-1^ dry weight). Each point stands for a mean ±SD (*n*=3). Correlation coefficient values between gene expression profile and anthocyanin levels are presented above each gene legend correspondingly (*n*=14, *r*_0.05_= 0.497, *r*_0.01_= 0.628).

To examine how the 34 DEGs in the WGCNA module ‘Pink’ may be expressed in more diverse apple phenotypes, the same ten representative genes used previously in Fig. 5 were selected and assayed using qRT-PCR in 14 *Malus* accessions of varying fruit colour characteristics (Fig. 9; Supplementary Table S2). Based on their colour in skin and flesh, the 14 *Malus* accessions could be categorized into the following four groups: 1, yellow skin/white flesh (Vogelcalville, Smoothgold, PRI 1345 and Blondee); 2, red skin/white flesh (Kidd’s D-8, GaleGala, Tydeman Red, Jerseymac, PRI 1236 and Earlibrite Delicious; 3, red skin/red flesh (Redflesh, Pink Wood and Eleyi); and 4, yellow skin/red flesh (Rose Bud). Expression of the ten genes was analysed in both skin and flesh tissues of the 14 accessions at immature (S2) and mature (S4) stages (Fig. 9).

In fruit skin tissues (Fig. 9A, B), anthocyanin contents (Supplementary Table S8) were markedly higher in red skin groups (groups 2 and 3) than in yellow skin groups (groups 1 and 4). Anthocyanin contents were also higher in S4 than in S2, especially in red skin groups. Correlation analysis (Supplementary Table S9) underscored that among the ten genes, two (*MdCHI* and *MdLDOX*) showed no significant correlation between expression and anthocyanin levels at both stages. However, the remainder eight genes showed significant correlation between expression and anthocyanin content at both S2 (*r*=0.667–0.835, *P*=0.0091–0.0002) and S4 (*r*=0.796–0.955, *P*=0.0006–1.1×10^–8^) (Fig. 9A, B).

In fruit flesh tissues (Fig. 9C, D), anthocyanin levels (Supplementary Table S8) were lower than in skin tissues in general. Between the white and red flesh groups (groups 1 and 2 vs. groups 3 and 4), the latter was obviously of higher anthocyanin than former. Increase of anthocyanin at S4 was also observed in groups 2, 3 and 4, especially the red flesh groups (3 and 4). Correlations analysis (Supplementary Table S9) demonstrated significant correlation in the ten genes between expression and anthocyanin content at S2 (*r*=0.820–0.972, *P*=0.0003–1.0×10^–8^) and S4 (*r*=0.824–0.969, *P*=0.0003–1.0×10^–8^).

The correlations between the expression of the ten genes and the concentration of the four individual anthocyanins detected in apple fruit tissues (Cy-3-galactoside, Cy-3-glucoside, Cy-3-arabinoside, and Cy-3-rutinoside) were similar to those observed for total anthocyanin (Fig. 9, Supplementary Table S9). However, none of the ten genes’ expressions correlated with the content of Cy-3-rutinoside in fruit skin tissue except for *MdGST* at S2 (*r*=0.545, *P*=0.0439). Overall, these data suggested that at least eight of the ten selected genes in the WGCNA module ‘Pink’ were also significantly correlated with anthocyanin contents in the 14 diverse apple accessions.

To determine how the expression profiles of the ten genes may explain the variation of fruit colour in skin and flesh, a hierarchical cluster analysis was performed (Supplementary Fig. S6). In fruit skin colour, the expression data divided the 14 accessions into two main clusters, which correspond well to the yellow and red fruits, except for ‘Rose Bud’ at S2 (Supplementary Fig. S6A, B). In fruit flesh, the expression levels of the ten genes precisely predicted the fruit flesh colour groups, i.e. white and red (Supplementary Fig. S6C, D). These data further suggested that the ten selected genes from the WGCNA module ‘Pink’ were involved in the anthocyanin biosynthesis and accumulation in the 14 apple accessions.

### Genetic mapping of the yellow fruit skin trait in progeny of ‘Royal Gala’

To genetically map the yellow fruit skin trait that might be associated with the yellow fruit mutation in BLO, the trait was observed in the mapping population derived from cross ‘Royal Gala’×PI 613988. Among the 188 genotyped seedling trees (Wang *et al.*, 2012), 162 bore fruit with 34 of yellow skin and 128 of non-yellow skin, i.e. red fruit, while the remainder 26 did not set fruit. The fruit colour segregations fit ratio 3(red):1(yellow) well (*P*=0.24, chi-square test), suggesting yellow fruit skin segregated as a recessive trait and was controlled by a single genetic locus. Using Kruskal-Wallis QTL mapping, the yellow fruit skin trait was revealed to link tightly with marker CN444542 on linkage group 9 in both parents (Supplementary Fig. S7), a region known to harbour *MdMYB10* (Chagné *et al.*, 2007; Morimoto *et al.*, 2013).

## Discussion

### Identification of the WGCNA module highly associated with anthocyanin and its implications

Somatic mutation in plants, including woody species, could arise from genetic (Kobayashi *et al.*, 2004; Venturi *et al.*, 2006; Nwafor *et al.*, 2014; Otto *et al.*, 2014) or epigenetic (Kvaalen and Johnsen, 2008) changes. These mutants, almost identical to their parents in genetic background, are considered desirable genetic material to study the novel traits of mutation (Nwafor *et al.*, 2014; Otto *et al.*, 2014). Somatic mutations causing fruit colour alteration have been recognized amongst several woody fruit species, including grape (Kobayashi *et al.*, 2004; Azuma *et al.*, 2009), pear (Thomas *et al.*, 2010; Wang *et al.*, 2013; Qian *et al.*, 2014), and apple (Ben-Yehudah *et al.*, 2005; Venturi *et al.*, 2006; Xu *et al.*, 2012). In this study, the transcriptomes of ‘Blondee’ (BLO), a yellow-skin apple fruit somatic mutant, and its red-skin parent ‘Kidd’s D-8’ (KID), commonly known as ‘Gala’, were characterized to identify genes that are expressed differentially between them, and thereby gain insights into the molecular mechanisms relevant for anthocyanin regulation, biosynthesis, transport and accumulation in apple.

Overall, 3299 differentially expressed genes (DEGs) were identified. These DEGs are considered to encompass the most relevant genes for anthocyanin as they include not only DEGs between the two ‘Gala’ strains at four developmental stages, but also DEGs between two adjacent developmental stages within BLO and/or KID, which account for colour progression (in KID) that may not be captured by direct comparison between BLO or KID. A weighted gene co-expression network analysis (WGCNA) of the 3299 DEGs enabled us to identify a WGCNA module ‘Pink’ of 34 genes highly correlated (*r*=0.95, *P*=9.0×10^–13^) with anthocyanin contents (Fig. 3; Table 1). This suggested that suppression of the module would result in the loss-of-colour in BLO.

Based on MapMan annotation, 24 (~71%) of the 34 member genes in module ‘Pink’ were found to be putatively associated with anthocyanin while the other ten were either of unknown function or of function not previously linked to anthocyanin. This highlights that the co-gene network analysis using the WGCNA package (Zhang and Horvath, 2005; Langfelder and Horvath, 2008), which has been used widely for similar analysis in other studies (Hollender *et al.*, 2014; Kogelman *et al.*, 2014; Miller *et al.*, 2014; Wang and Huang, 2014), was meaningful in the biological sense in this study.

The 24 genes known of putative roles in anthocyanin cover many steps in the pathway, and function as regulatory, biosynthetic or transport components (Table 1; Fig. 4). Notably, 12 of the 24 genes were characterized previously, whereas the other 12 were reported only in this study. Examining ten representative genes in 14 *Malus* accessions of diverse fruit colour phenotypes demonstrated that at least eight were expressed in significant correlation with anthocyanin contents in both skin and flesh tissues, suggesting most member genes in module ‘Pink’, if not all, may play a role in anthocyanin, irrespective of their annotation by MapMan. This seemed to be consistent with the observations that most genes in the anthocyanin pathway function in concert to determine anthocyanin levels in apple (Honda *et al.*, 2002), which differs from pear and grape where the UFGT enzyme has been shown to be the key regulatory step in anthocyanin biosynthesis (Boss *et al.*, 1996; Kobayashi *et al.*, 2004; Wang *et al.*, 2013). Since 22 of the 34 genes in the WGCNA module were not characterized, this study provides not only new insights into the anthocyanin pathway, but also a list of interesting candidate genes for more dedicated functional studies in the future.

### Perspectives of the role of *MdGST* and its regulation

The transcripts of *MdGST* were suppressed most dramatically during BLO fruit development. In plants, glutathione *S*-transferases (GSTs) act as non-enzymatic carrier proteins (ligandins), which escort endogenous compounds to the transporters at tonoplast (Edwards *et al.*, 2000). In the anthocyanin pathway, GST proteins perform at the last step through the conjugation of glutathione to anthocyanins. Subsequently, the conjugated anthocyanins will be transported, through the recognition of glutathione tag molecules, by a tonoplast-localized ATP-binding cassette pump into the vacuole, the permanent storage compartment of anthocyanin (Alfenito *et al.*, 1998; Sun *et al.*, 2012). Gene knockout and complementation studies have demonstrated that GSTs are indelibly involved in anthocyanin transport. A *Zea mays* knockout mutant of a single *GST* (*Bz2*) produces yellow skin kernels due to disabled transport of anthocyanin into vacuole coupling with anthocyanin accumulation in cytosol (Mueller *et al.*, 2001; Goodman *et al.*, 2004). Moreover, transient expression of *Bz2* or a petunia GST (*A9*) in a *Bz2*-deficient maize mutant was able to complement the *Bz2* deficiency, resulting in kernels with red spots due to anthocyanin accumulation in the vacuole (Marrs *et al.*, 1995; Alfenito *et al.*, 1998). Interestingly, BLO fruit mutants seemingly behaved similarly to the maize *Bz2*-deficient mutant in terms of suppression of *GST*, which gives rise to yellow fruit. However, our sequence analysis of *MdGST* as well as methylation survey of its promoter regions did not show noticeable variations that can account for the sharply contrasting *MdGST* expression profiles of BLO and KID, suggesting the existence of a transcription repression mechanism of *MdGST* in BLO.

It is known that anthocyanin accumulation is coordinated via the *bHLH*/*MYC* and *MYB* regulatory factors that control anthocyanin-related genes expression through binding to specific DNA elements in the promoter region of their target genes (Ban *et al.*, 2007; Espley *et al.*, 2009; An *et al.*, 2012). Further, ectopic expression of *VvMYBA1* in grape triggered *de novo* production and storage of anthocyanins partially due to transcription activation of a downstream *VvGST* isogen (Cutanda-Perez *et al.*, 2009). Interestingly, sequence analysis revealed that the *MdGST* promoter possesses 19 *MYB* and 11 *MYC cis*-acting regulatory elements that have been proven in several plant species (Solano *et al.*, 1995; Bodeau and Walbot, 1996; Hartmann *et al.*, 2005; Wang *et al.*, 2013). It was therefore suggested that the transcription of *MdGST* is controlled by *MYB* and/or *bHLH/MYC* transcription factors.

RNA-seq data analysis detected eight differentially expressed *bHLH* homologues in the 3299 DEGs, but none of them were members of the WGCNA module ‘Pink’, indicating that *bHLH* transcription factors is less likely to be responsible for the distinct fruit colour trait between the BLO and KID. However, *MdMYB10* was identified as one of the 34 DEGs in the ‘Pink’ module. In addition, genetic analysis in the progeny of ‘Royal Gala’ mapped the yellow fruit skin trait to a region (close to marker CN444542) known to harbour *MdMYB10* on linkage group 9 (Chagné *et al.*, 2007; Morimoto *et al.*, 2013), suggesting the possibility of *MdMYB10* functioning as the genetic determinant for fruit colour in ‘Gala’. These data implicated that suppression of *MdGST* through down-regulation of *MdMYB10* is likely an important mechanism elucidating the loss-of-colour in BLO.

### Epigenetic regulation of *MdMYB10* in ‘Blondee’ and its developmental control

The aberrant expression of the master anthocyanin regulator *MYB*, and consequently the disruption of anthocyanin accumulation, could be caused by sequence mutations in the *MYB* coding or promoter regions (Kobayashi *et al.*, 2004; Espley *et al.*, 2009; Chiu *et al.*, 2010; Hichri *et al.*, 2011), or by DNA methylation or demethylation in the promoter (Cocciolone and Cone, 1993; Sekhon and Chopra, 2009; Telias *et al.*, 2011; Xu *et al.*, 2012; Wang *et al.*, 2013). This study did not detect any difference in the *MdMYB10* sequences between BLO and KID. However, methylation analysis of the *MdMYB10* promoter revealed that methylation levels in two regions (MR3 and MR7) were inversely correlated with red fruit coloration in KID, BLO and ‘GaleGala’, i.e. hyper methylation is associated with low anthocyanin in BLO while low methylation is associated with high anthocyanin in KID and ‘GaleGala’. For MR3, a similar region has been identified in apple and pear (Telias *et al.*, 2011; Wang *et al.*, 2013), but the relevance of methylation in the MR7 region has not been reported until this study. Compared with other studies (Telias *et al.*, 2011; Wang *et al.*, 2013), the differences in methylation levels between BLO and KID were enormous (Fig. 7), which reflected well the contrast coloration between the two ‘Gala’ stains: a nearly complete loss of anthocyanin in BLO vs. a normal red coloration in KID.

All three cytosine contexts (CG, CHG and CHH) in the MR3 region exhibited higher methylation levels in BLO than in KID and ‘GaleGala’. Similar hyper-methylation in the three types of cytosines in the promoter of *PcMYB10* has also been observed in pear (Wang *et al.*, 2013). However, in maize, the type of DNA methylation that induced colour variation was largely due to the methylation of CG/CHG cytosines (Sekhon and Chopra, 2009). In the MR7 region, the methylation in the two cytosine contexts (CG and CHG) did not show a significant difference between BLO and KID, but the level of CHH cytosine methylation in fruit skin was dramatically higher in BLO than in KID and ‘GaleGala’. Such a pattern of methylation in MR7 appeared to be rare, if not unique. Since MR7 is located more upstream than MR3 and the methylation in MR7 was correlated equally negatively with anthocyanin content and the expression of *MdMYB10* and *MdGST* as that of the MR3 region, it would be interesting to investigate the methylation behaviour in MR7 more systematically in the future to further illustrate the epigenetic role of MR7, together with MR3, in regulation of *MdMYB10*.

Several environmental stimulators, such as light and low temperature, have been experimentally recognized to impact the expression of *MYB* (Sekhon and Chopra, 2009; Takos *et al.*, 2006). MYB protein can also activate the transcription of *MYB* through auto-regulatory feedforward mechanisms of the promoter (Espley *et al.*, 2009). Interestingly, the highly differentially methylated MR3 region of the *MdMYB10* promoter comprises numerous *cis*-acting elements, including *MYB*-, *MYC*-binding sites, light-responsive elements, and low temperature-responsive elements, which may potentially be involved in *MdMYB10* up-regulation (Supplementary Table S6). This suggested that the methylation within the MR3 region may reduce not only the binding of proteins MYB and MYC, but also the impact of the environmental stimulators. Several recent reports suggested the positive regulatory mechanism of jasmonic acid in anthocyanin accumulation through upstream control of the MBW complex activity (Qi *et al.*, 2011; Qian *et al.*, 2014). However, this mechanism appears to be limited to explaining the regulation of anthocyanin accumulation under specific stressful circumstances. In this study, DNA methylation in MR3 and MR7 is clearly a mechanism regulating *MdMYB10*, the master regulator of the anthocyanin pathway in apple.

Moreover, methylations in the MR3 and MR7 regions were observed to gradually increase along with fruit development and mutation, suggesting methylation in the promoter of *MdMYB10* is developmentally controlled. Such change in methylation activity in a fruit development-dependent manner in the promoter of *MdMYB10* has not been reported elsewhere to the best of our knowledge, and thus adds a new level of complexity to the mechanisms that regulate the fruit colour trait.

## Conclusions

Through a comparative transcriptome analysis of yellow fruit mutant ‘Blondee’ and its parent ‘Kidd’s D-8’ (also known as ‘Gala’), at four stages of fruit development and maturation, the loss-of-colour of ‘Blondee’ is concluded likely to be the consequence of collective repression of the 34 genes, which are members of a WGCNA module ‘Pink’ that was highly associated with fruit anthocyanin content in this study. Based on the expression profile of ten of the 34 genes examined in 14 Malus accessions of varying skin and/or flesh colour, most genes, if not all, in the WGCNA module ‘Pink’ are viewed to play a role in anthocyanin accumulation in apple. These results demonstrated the power of RNA-seq based transcriptome analysis in conjunction with WGCNA in identification of a co-expression gene network associated with the anthocyanin. Although 12 of the 34 genes in module ‘Pink’ were previously characterized and known to have roles in anthocyanin accumulation, the remainder 22 are novel anthocyanin-associated genes first reported in the present study. This novel set of genes provides not only new insights into the anthocyanin pathway, but also important clues for more dedicated studies to broaden our knowledge of the anthocyanin pathway in apple. The detailed sequence and methylation analyses of MdMYB10 and MdGST, which are among the most suppressed genes in ‘Blondee’, demonstrated that the starkly differentiated methylation activities in the promotor regions (MR3 and MR7) of MdMYB10, the transcription factor known as the master regulator of anthocyanin in apple, are likely epigenetic factors causing the fruit colour variation between ‘Blondee’ and ‘Kidd’s D-8’.

## Supplementary data

Supplementary data are available at *JXB* online.

Supplementary Fig. S1. Overview of the trees and fruit of ‘Kidd’s D-8’ and ‘Blondee’.

Supplementary Fig. S2. HPLC chromatogram traces of sample Jerseymac’s mature skin.

Supplementary Fig. S3. Evaluation of the KID and BLO parent-mutant relationships.

Supplementary Fig. S4. Venn diagram representation of the number of all expressed genes identified by RNA-seq analysis in the skin tissues of BLO and KID fruit at varying developmental stages.

Supplementary Fig. S5. Screening of MdGST promoter regions for different methylation levels between KID and BLO using McrBC-PCR.

Supplementary Fig. S6. Hierarchical cluster analysis (Ward’s method) of fruit scan and flesh samples from 14 *Malus* accessions.

Supplementary Fig. S7. Kruskal-Wallis QTL mapping of the yellow-scan fruit trait in ‘Royal Gala’.

Supplementary Table S1. List of oligonucleotide primers and sequences.

Supplementary Table S2. Developmental stages and fruit color characteristics of apple genotypes.

Supplementary Table S3. Overview of mapping of RNA-seq reads.

Supplementary Table S4. Anthocyanin type and content in the skin of two ‘Gala’ strains KID and BLO during fruit development.

Supplementary Table S5. Comparison of the anthocyanin related regulatory factors, and biosynthesis and transport genes identified in this study with those reported previously.

Supplementary Table S6. Promoter analysis using PLACE Signal Scan Search database.

Supplementary Table S7. Correlations between the MdMYB10 promoter methylation and anthocyanin contents and gene expression.

Supplementary Table S8. Anthocyanin contents in the skin and flesh tissues of immature and mature fruit of 14 *Malus* accessions.

Supplementary Table S9. Pearson’s correlation coefficient between the expression profile of ten selected genes and the contents of total anthocyanin and individual anthocyanins.

Supplementary Data

## Abbreviations:

PAL: phenylalanine ammonia lyase
C4H: cinnamate 4-hydroxylase
4CL: 4-coumarate CoA ligase
HCT/HQT: quinate hydroxycinnamoyl/hydrxycinnamoyl CoA shikimate
C3H: 4-coumarate 3-hydroxylase
CHS: chalcone synthase
CHI: chalcone isomerase
F3H: flavone 3-hydroxylase
F3’H: flavonoid 3’-hydroxylase
FLS: flavonol synthase
DFR: dihydroflavonol reductase
LAR: leucoanthocyanidin reductase
LDOX: leucoanthocyanidin dioxygenase
ANR: anthocyanidin reductase
UFGT: UDP-glucose: flavonoid 3-O-glucosyltransferase
GST: glutathione *S*-transferase
MATE: multidrug and toxic compound extrusion transporters
BLO: Blondee
KID: Kidd’s D-8
MBW: MYB-bHLH-WD40 complex
DEGs: differentially expressed genes
WGCNA: weighted gene co-expression network analysis.
