# Supplementary material for: Transcriptome analysis of an apple (Malus × domestica) yellow fruit somatic mutation identifies a gene network module highly associated with anthocyanin and epigenetic regulation
Source: J Exp Bot. 2015 Sep 28;66(22):7359–76. doi: 10.1093/jxb/erv433 (PMC4765799; doi:10.1093/jxb/erv433)
Supplement: Supplementary Data [file supp_66_22_7359__index.html]

Transcriptome analysis of an apple (Malus × domestica) yellow fruit somatic mutation identifies a gene network module highly associated with anthocyanin and epigenetic regulation — Transcriptome analysis of an apple (Malus × domestica) yellow fruit somatic mutation identifies a gene network module highly associated with anthocyanin and epigenetic regulation — Supplementary Data 

# Transcriptome analysis of an apple (*Malus* × *domestica*) yellow fruit somatic mutation identifies a gene network module highly associated with anthocyanin and epigenetic regulation

## Supplementary Data

Data files

- Supplementary Data - Supplementary Data
