# Supplementary material for: Transcriptome analysis of an apple (Malus × domestica) yellow fruit somatic mutation identifies a gene network module highly associated with anthocyanin and epigenetic regulation
Source: J Exp Bot. 2015 Sep 28;66(22):7359–76. doi: 10.1093/jxb/erv433 (PMC4765799; doi:10.1093/jxb/erv433)
Supplement: Supplementary Data [file supp_erv433_jexbot155267_file001.pdf]

Supplementary Data

Title: Transcriptome analysis of an apple (*Malus* ×*domestica*) yellow fruit somatic mutation identifies a gene network module highly associated with anthocyanin and epigenetic regulation

Short Running Title: Transcriptome characterization of an apple yellow fruit mutation

Islam El-Sharkawy[1,2], Dong Liang[1,2,3] and Kenong Xu[1*].

[1]Horticulture Section, School of Integrative Plant Science, Cornell University, NYSAES, Geneva, NY 14456, USA.
[2]These authors contributed equally to this work
[3]Present address: Institute of Pomology & Olericulture, Sichuan Agricultural University, Chengdu, Sichuan 611130, China.

***Corresponding author**: Kenong Xu
Address: Horticulture Section, School of Integrative Plant Science, Cornell University, New York State Agricultural Experiment Station, Geneva, NY 14456, USA
Telephone: .315-787-2496
Fax: 315-787-2216
E-mail: kx27@cornell.edu

Supplementary Tables

- Table S1. List of oligonucleotide primers and sequences
- Table S2. Developmental stages and fruit color characteristics of apple genotypes
- Table S3. Overview of mapping of RNA-seq reads
- Table S4. Anthocyanin type and content in the skin of two 'Gala' strains KID and BLO during fruit development
- Table S5: Comparison of the anthocyanin related regulatory factors, and biosynthesis and transport genes identified in this study with those reported previously.
- Table S6. Promoter analysis using PLACE Signal Scan Search database
- Table S7. Correlations between the *MdMYB10* promoter methylation and anthocyanin contents and gene expression
- Table S8. Anthocyanin contents in the skin and flesh tissues of immature and mature fruit of 14 *Malus* accessions
- Table S9. Pearson's correlation coefficient between the expression profile of ten selected genes and the contents of total anthocyanin and individual anthocyanins

Supplementary Figures

- Fig. S1. Overview of the trees and fruit of 'Kidd's D-8' and 'Blondee'. (
- Fig. S2. HPLC chromatogram traces of sample Jerseymac's mature skin. Peaks were annotated with retention time and individual anthocyanin name. mAU: milli absorption units.
- Fig. S3. Evaluation of the KID and BLO parent-mutant relationships. (
- Fig. S4. Venn diagram representation of the number of all expressed genes identified by RNA-seq analysis in the skin tissues of BLO and KID fruit at varying developmental stages.

- Fig. S5. Screening of *MdGST* promoter regions for different methylation levels between KID and BLO using McrBC-PCR.
- Fig. S6. Hierarchical cluster analysis (Ward's method) of fruit scan and flesh samples from 14 *Malus* accessions.
- Fig. S7. Kruskal-Wallis QTL mapping of the yellow-scan fruit trait in 'Royal Gala'

Supplementary References

Brueggemann J, Weisshaar B, Sagasser M. 2010. A WD40-repeat gene from *Malus x domestica* is a functional homologue of Arabidopsis thaliana *TRANSPARENT TESTA GLABRA1*. *Plant Cell Reports* **29,** 285-294.

Davies KM. 1993. A cDNA clone for flavanone 3-hydroxylase from *Malus*. *Plant Physiology* **103,** 291.

Espley RV, Hellens RP, Putterill J, Stevenson DE, Kutty-Amma S, Allan AC. 2007. Red colouration in apple fruit is due to the activity of the MYB transcription factor, MdMYB10. *The Plant Journal* **49,** 414-427.

Fischer TC, Halbwirth H, Meisel B, Stich K, Forkmann G. 2003. Molecular cloning, substrate specificity of the functionally expressed dihydroflavonol 4-reductases from *Malus domestica* and *Pyrus communis* cultivars and the consequences for flavonoid metabolism. *Archives of Biochemistry and Biophysics* **412,** 223-230.

Fischer TC, Halbwirth H, Roemmelt S, Sabatini E, Schlangen K, Andreotti C, Spinelli F, Costa G, Forkmann G, Treutterc D, Stichb K. 2006. Induction of polyphenol gene expression in apple (*Malus x domestica*) after the application of a dioxygenase inhibitor. *Physiologia Plantarum* **128,** 604-617.

Halbwirth H, Fischer TC, Schlangen K, Rademacher W, Schleifer KJ, Forkmann G, Stich K. 2006. Screening for inhibitors of 2-oxoglutarate-dependent dioxygenases: Flavanone 3β-hydroxylase and flavonol synthase. *Plant Science* **171,** 194-205.

Han Y, Vimolmangkang S, Soria-Guerra RE, Rosales-Mendoza S, Zheng D, Lygin AV, Korban SS. 2010. Ectopic expression of apple *F3'H* genes contributes to anthocyanin accumulation in the *Arabidopsis tt7* mutant grown under nitrogen stress. *Plant Physiology* **153,** 806-820.

Hellens RP, Allan AC, Friel EN, Bolitho K, Grafton K, Templeton MD, Karunairetnam S, Gleave AP, Laing WA. 2005. Transient expression vectors for functional genomics, quantification of promoter activity and RNA silencing in plants. *Plant Methods* **1,** 13.

Henry-Kirk RA, McGhie TK, Andre CM, Hellens RP, Allan AC. 2012. Transcriptional analysis of apple fruit proanthocyanidin biosynthesis. *Journal of Experimental Botany* **63,** 5437-5450.

Honda C, Kotoda N, Wada M, Kondo S, Kobayashi S, Soejima J, Zhang Z, Tsuda T, Moriguchi T. 2002. Anthocyanin biosynthetic genes are coordinately expressed during red coloration in apple skin. *Plant Physiology and Biochemistry* **40,** 955-962.

Kim SH, Lee JR, Hong ST, Yoo YK, An G, Kim SR. 2003. Molecular cloning and analysis of anthocyanin biosynthesis genes preferentially expressed in apple skin. *Plant Science* **165,** 403-413.

Li H, Flachowsky H, Fischer TC,·Hanke MV, Forkmann G, Treutter D,·Schwab W, Hoffmann T, Szankowski I. 2007. Maize *Lc* transcription factor enhances biosynthesis of

anthocyanins, distinct proanthocyanidins and phenylpropanoids in apple (*Malus domestica* Borkh.). *Planta* **226,** 1243-1254.
Takos AM, Ubi BE, Robinson SP, Walker AR. 2006. Condensed tannin biosynthesis genes are regulated separately from other flavonoid biosynthesis genes in apple fruit skin. *Plant Science* **170,** 487-499.
Venisse JS, Malnoy M, Faize M, Paulin JP, Brisset MN. 2002. Modulation of defense responses of *Malus* spp. during compatible and incompatible interactions with *Erwinia amylovora*. *Molecular Plant-Microbe Interaction* **15,** 1204-1212.

Table S1. List of oligonucleotide primers and sequences

Category	Name	Oligonucleotide sequence	Notes /Region/Gene ID
SSR primers for fingerprinting	CH01g05(F)	CATCAGTCTCTTGCACTGGAAA	Informative
	CH01g05(R)	GACAGAGTAAGCTAGGGCTAGGG	
	CH01h01(F)	GAAAGACTTGCAGTGGGAGC	Informative
	CH01h01(R)	GGAGTGGGTTTGAGAAGGTT	
	CH02b07(F)	CCAGACAAGTCATCACAACACTC	Informative
	CH02b07(R)	ATGTCGATGTCGCTCTGTTG	
	CH02g01(F)	GATGACGTCGGCAGGTAAAG	Informative
	CH02g01(R)	CAACCAACAGCTCTGCAATC	
	Hi12c02(F)	GCAATGGCGTTCTAGGATTC	Informative
	Hi12c02(R)	GTTTCACCAACAGCTGGGACAAG	
	C526(F)	CGATACGAGTGGGTTCGATT	Informative
	C526(R)	CTGGCGAAGAACGGAACTTA	
	C2444(F)	GCATGAACCTCTCCACCTC	Informative
	C2444(R)	GGTTTGCAGAAAAGGACTGC	
	C3656(F)	AATGGGTCGCAAACTCAAAG	Informative
	C3656(R)	TGGCTCCTGATTATTTTCGG	
	CH01g12(F)	CCCACCAATCAAAAATCACC	Informative
	CH01g12(R)	TGAAGTATGGTGGTGCGTTC	
	CH01h10(F)	TGCAAAGATAGGTAGATATATGCCA	Informative
	CH01h10(R)	AGGAGGGATTGTTTGTGCAC	
	CH02f06(F)	CCCTCTTCAGACCTGCATATG	
	CH02f06(R)	ACTGTTTCCAAGCGATCAGG	
	CH02g09(F)	TCAGACAGAAGAGGAACTGTATTTG	Informative
	CH02g09(R)	CAAACAAACCAGTACCGCAA	
	C509(F)	TCTTCACACCCTTCAATCCC	
	C509(R)	GGAGAGCTGAAGAGCCAAGA	
	C2047(F)	GGTCTTCATCAAGCACGGTT	
	C2047(R)	GCTCACTTTCCCACCCATTA	
	C2630(F)	CCTCCATTTACAACCAAAGGG	
	C2630(R)	CAGCTTTTTCTGTCCGAAGG	
	C4251(F)	TAAGGGACAAACCTCCTCCC	
	C4251(R)	GGTGGGGTTGAAAGATGAGA	
MdMYB10 promoter isolation and McrBC-PCR analysis	MR1(F)	GGTATCTTATGGTGGTCAAAGATG	-440 to +1
	MR1(R)	CTTATCTGCTAGCAGCTAAGCTTA	
	MR2(F)	CTGAGATTGACTCTTGTGAAAGCT	-856 to -383

MR2(R)	GTGAATGCAGAATCGTGTAACTAGT	
MR3(F)	TTAACGGAATCCAACGAAGACAAG	-1246 to -780
MR3(R)	GCTACACCTAACACATTGCTCAAT	
MR4(F)	TGAGATAGGTCCGGTTCTATTTCT	-1657 to -1184
MR4(R)	ATCCCTTTCCCTTATTTGTTCCGT	
MR5(F)	CAATTGCAGTGCTCAGAAATCGTT	-2044 to -1590
MR5(R)	CCGGACCCGTTTATAAATAGAACA	
MR6(F)	CCATTTCCACCGTTCATTTCTAAG	-2255 to -1872
MR6(R)	AACAGCAAACACCCAAAATCCCTT	
MR7(F)	CACTAGCTTCGGATTCCTTAGGA	-2585 to -2117
MR7(R)	CGGTTTAGTTTCTGGGAATTCACA	

To be continued

Table S1. List of oligonucleotide primers and sequences (continued)

Category	Name	Oligonucleotide sequence	Notes /Region/Gene ID
Bisulfite sequencing of *MdMYB10 promoter*	MR3z(F)	TTATGTATAAGAATTTGGGGTTTTGGA	
	MR3z(R)	CTACACCTAACACATTACTCAATATAA	
	MR5z(F)	GTAATTATAATTATGATTTTTGGGGTAG	
	MR5z(R)	CRAACTCAAAATTAAAAAAACCAAACC	
	MR7z(F)	YGGATTTTTTAGGAATTTTGAAGTTAAG	
	MR7z(R)	AACAACAAACACCCAAAATCCCTTTAA	
MdGST promoter isolation and McrBC-PCR analysis	GST1(F)	CAGAGGGAGACTGAAATGCCTAT	-460 to +116
	GST1(R)	TCTCCTGCCTCAAGATCAACATC	
	GST2(F)	CCATTGTATTTCTAAGAGGGAGCA	-995 to -398
	GST2(R)	AAAGGGACCAACTTGTCGTTTCG	
	GST3(F)	AACGAAACCCAGTGTATTCCGCA	-858 to -1588
	GST3(R)	ATGCACTTTCCTTCCAGCAATCC	
	GST4(F)	CCTAGAACGATACCCTACTTACAT	-1436 to -2070
	GST4(R)	ACACGATGAAATTTACTTTCTGCCA	
	GST5(F)	GTTGCTAGTCACAATCACGTTAAG	-1974 to -2725
	GST5(R)	CTCGTACAGAGATGATGCGTAAAA	
qRT-PCR Primers	Act(FQ)	TGAACCCAAAGGCTAATCG	M921834
	Act(RQ)	GGGAAAGAACAGCCTGGAT	
	MYB(FQ)	AGGCAGTGCGTTGAGATTC	M127691
	MYB(RQ)	CAGCTCTTCCTGCACCTGT	
	WD(FQ)	GCCATTTCATGGTCTCCAG	M129392
	WD(RQ)	TCCAGGTCTCACCACATCC	
	GHS(FQ)	CCGCTGAGAAAGGACTCAA	M686661
	GHS(RQ)	AACGGTCTCGACAGTGAGG	
	CHI(FQ)	GTCAAGGAATTACCGACATTGA	M252589
	CHI(RQ)	CACAACTTCAGGGTCCAAGTAA	
	F3H(FQ)	CTGGGGTATTTTCCAGATTGTT	G104117
	F3H(RQ)	TGGCAAAGCAAAGAACTCTCTA	
	F3'H(FQ)	CAACAAGAGCTGGACCAAGTAG	M190489
	F3'H(RQ))	TTACTGCTTGGAGGTAGGTCAA	
	DFR(FQ)	GAGCAAGCTGCATGGAAAT	M494976
	DFR(RQ))	CATGAGAAATGGCCCAATC	
	LDOX(FQ)	TGAGCCACCAAAGGAGAAG	M360447
	LDOX(RQ))	AAAGTTCGTGGTGGGAACA	
	UFGT(FQ)	TGCAGACCAGAGGCTTAATG	M478252
	UFGT(RQ))	CCTCCCTGGTAAAAACTCCA	
	GST(FQ)	GAGCCATCTTCCTGGGATT	M252292
	GST(RQ))	CCCACCATGCATTCACTTT	

Table S2. Developmental stages and fruit color characteristics of apple genotypes

Cultivar	Taxonomy	Stage	Sampling date (m/d/y)	DAFB [a]	Skin/Flesh Color
Kidd's D-8	*M. domestica*	S1	7/16/2013	85	Yellow /White
		S2	8/7/2013	107	
		S3	8/28/2013	128	
		S4	9/14/2013	145	
Blonde	*M. domestica*	S1	7/16/2013	85	Red/White
		S2	8/7/2013	107	
		S3	8/28/2013	128	
		S4	9/14/2013	145	
Vogelcalville	*M. domestica*	S2	8/29/2014	130	Yellow/White
		S4	10/1/2014	162	
Smoothgold	*M. domestica*	S2	9/3/2014	135	Yellow /White
		S4	10/8/2014	169	
PRI 1345	*M. hybrid*	S2	8/7/2014	107	Yellow /White
		S4	9/3/2014	135	
Blonde	*M. domestica*	S2	8/10/2014	110	Yellow /White
		S4	9/15/2014	146	
Rose Bud	*M. domestica*	S2	9/10/2014	141	Yellow-Red/Red
		S4	10/6/2014	167	
Kidd's D-8	*M. domestica*	S2	8/10/2014	110	Red/White
		S4	9/15/2014	146	
Galgala	*M. domestica*	S2	8/10/2014	110	Red/White
		S4	9/15/2014	146	
Tydeman Red	*M. domestica*	S2	8/8/2014	108	Red/White
		S4	8/29/2014	130	
Jerseymac	*M. domestica*	S2	8/7/2014	107	Red/White
		S4	9/1/2014	132	
PRI 1236	*M. hybrid*	S2	8/7/2014	107	Red/White
		S4	9/3/2014	135	
Earlibrite Delicious	*M. domestica*	S2	8/12/2014	112	Red/White
		S4	9/20/2014	151	
Red Flesh	*M. hybrid*	S2	8/7/2014	108	Red/Red
		S4	9/5/2014	136	
Pink Wood	*M. hybrid*	S2	8/7/2014	108	Red/Red
		S4	9/10/2014	141	
Eleyi	*M. hybrid*	S2	8/7/2014	108	Red/Red
		S4	9/3/2014	134	

[a] DAFB refers to days after full bloom. In 2014 S2 and S4 refer to sampling stages when fruit were immature and mature, respectively.

Table S3. Overview of mapping of RNA-seq reads

Sample name	Reads Passed Pipeline Casava 1.8	Total reads for mapping after depletion of rRNA reads	Mapped reads	Mapped reads (%)	Uniquely mapped reads	Uniquely mapped reads (%)
**S1-BLO**	**14,979,030±567,503**	**14,645,020±726,712**	**10,413,447±1,038,711**	**71.01±4.36**	**8,642,167±687,620**	**58.96±2.05**
S1-BLO1	14,331,274	13,808,859	9,318,562	67.48	7,860,447	56.92
S1-BLO2	15,388,745	15,001,973	11,384,966	75.89	9,153,474	61.02
S1-BLO3	15,217,072	15,124,229	10,536,813	69.67	8,912,579	58.93
**S1-KID**	**14,348,868±304,077**	**13,956,939±199,609**	**9,985,279±365,562**	**71.53±1.62**	**8,196,252±24,544**	**58.73±1**
S1-KID1	14,538,519	14,179,524	10,406,972	73.39	8,172,559	57.64
S1-KID2	14,509,948	13,793,822	9,758,102	70.74	8,221,567	59.6
S1-KID3	13,998,138	13,897,472	9,790,763	70.45	8,194,630	58.96
**S2-BLO**	**16,243,503±3,578,949**	**14,163,395±1,630,064**	**9,379,959±990,424**	**66.31±2.89**	**7,848,435±831,241**	**55.48±2.48**
S2-BLO1	20,289,683	16,045,356	10,439,342	65.06	8,734,231	54.43
S2-BLO2	14,948,561	13,194,586	8,477,144	64.25	7,085,403	53.7
S2-BLO3	13,492,265	13,250,242	9,223,390	69.61	7,725,672	58.31
**S2-KID**	**11,222,204±237,134**	**10,528,672±375,060**	**6,945,567±175,762**	**65.99±1.37**	**5,817,058±145,404**	**55.27±1.08**
S2-KID1	11,495,767	10,285,388	6,948,177	67.55	5,810,697	56.49
S2-KID2	11,075,177	10,340,024	6,768,515	65.46	5,674,939	54.88
S2-KID3	11,095,668	10,960,604	7,120,010	64.96	5,965,539	54.43
**S3-BLO**	**12,516,010±1,080,939**	**11,479,921±940,345**	**8,825,166±877,687**	**76.8±1.35**	**7,369,508±679,315**	**64.16±0.73**
S3-BLO1	11,698,267	11,264,330	8,645,527	76.75	7,253,060	64.39
S3-BLO2	13,741,522	12,509,340	9,778,775	78.17	8,099,519	64.75
S3-BLO3	12,108,241	10,666,093	8,051,197	75.48	6,755,944	63.34
**S3-KID**	**14,655,221±2,213,121**	**13,668,871±1,806,621**	**10,569,352±1,625,049**	**77.17±1.83**	**8,821,506±1,433,950**	**64.35±2.19**
S3-KID1	12,140,863	11,788,868	8,850,575	75.08	7,289,477	61.83
S3-KID2	15,516,918	13,825,884	10,776,715	77.95	9,043,573	65.41
S3-KID3	16,307,882	15,391,862	12,080,767	78.49	10,131,468	65.82
**S4-BLO**	**11,593,367±1,596,403**	**11,106,439±1,379,967**	**8,214,436±917,544**	**74.04±1.67**	**6,917,673±772,148**	**62.35±1.38**
S4-BLO1	10,019,687	9,736,235	7,223,357	74.19	6,085,362	62.5
S4-BLO2	11,548,852	11,087,117	8,385,616	75.63	7,056,960	63.65
S4-BLO3	13,211,562	12,495,966	9,034,335	72.3	7,610,697	60.91
**S4-KID**	**13,610,314±2,500,668**	**13,093,228±2,053,131**	**9,762,526±1,411,199**	**74.66±0.89**	**8,217,648±1,186,574**	**62.84±0.74**
S4-KID1	16,447,702	15,404,742	11,354,284	73.71	9,553,853	62.02
S4-KID2	12,655,587	12,393,564	9,268,664	74.79	7,811,983	63.03
S4-KID3	11,727,654	11,481,377	8,664,631	75.47	7,287,107	63.47
**Total**	**327,505,554**	**307,927,457**	**222,287,199**		**185,490,740**	
Mean	13,646,065	12,830,311	9,261,967	72.19	7,728,781	60.27
SD	2,288,952	1,851,922	1,450,153	4.53	1,183,954	3.76

Table S4. Anthocyanin type and content in the skin of the two 'Gala' strains KID and BLO during fruit development.

Devel. stage	Anthocyanin content (µg g^{-1} DW)											
	BLO						KID					
	C-3-Gal	C-3-Glu	C-3-Ara	Ukw nonacylated	Ukw acylated	Total	C-3-Gal	C-3-Glu	C-3-Ara	Ukw nonacylated	Ukw acylated	Total
S1	3 ±0.6	0	0	0	0	3 ±0.6	9.2 ±1.6	0	0	0	0	9.2 ±1.6
S2	12.2 ±2.3	0	0	0	0	12.2 ±2.3	39.2 ±3.3	0	0	0	0	39.2 ±3.3
S3	6.9 ±1.4	0	0	0	0	6.9 ±1.4	115.9 ±13.8	3.2 ±0.5	6.7 ±0.9	4.8 ±0.6	6.7 ±0.6	137.2 ±16.3
S4	5.3 ±1	0	0	0	0	5.3 ±1	346.3 ±61.7	5.4 ±0.7	26.7 ±3	12.2 ±1.4	16.2 ±2.6	406.7 ±68.8

Anthocyanins were quantified using malvidin-3- glucoside as standard.

C-3-Gal, C-3-Glu, C-3-Ara, Ukw nonacylated, and Ukw acylated refer to cyanidin 3-galactoside, cyanidin 3-glucoside, cyanidin 3-arabinoside, unknown nonacylated, and unknown acylated anthocyanins, respectively.

KID and BLO refer to Kidd’s D-8 and Blondee apple strains, respectively.

Developmental stages: S1: 7/16/2013; S2: 08/07/2013; S3: 08/28/2013; S4: 09/14/2013

Table S5: Comparison of the anthocyanin related regulatory factors, biosynthesis and transport genes identified in this study with those reported previously.

Gene ID	Gene name	Chr. location	This study	Previously Reported	
				Accession No.	Reference
M259614	MdMYB10	9	YES	DQ267896	Espley *et al.*, 2007
M127691		9	YES	-	-
M906307	MdWD40	1	NO	GU173813, GU173814	Brueggemann *et al.*, 2007
M129392		14	YES	-	-
M225698	MdC4H	unanchored	NO	EB135197	Henry-Kirk *et al*., 2012
M576346		3	YES	-	-
M287029	MdC3H	8	YES	-	-
M428573		8	YES	-	-
M126567	MdCHS	unanchored	YES	AF494401, X68977	Venisse *et al.*, 2002; Fischer *et al.*, 2006
M575740		9	YES	DQ026297, AF494402	Venisse *et al.*, 2002; Hellens *et al.*, 2005
M686661		9	YES	-	-
M686666		9	YES	AB074485	Honda *et al.*, 2002
M682953	MdCHI	1	NO	AF494398	Venisse *et al.*, 2002
M684169		11	NO	AF494399	Venisse *et al.*, 2002
M205890		11	NO	AF494400	Venisse *et al.*, 2002
M759336		14	NO	X68978	Takos *et al.*, 2006a
M252589		12	YES	-	-
M134791		4	YES	-	-
G104117	MdF3H	2	YES	AF117270, AY965339, AY965340, X69664	Davies, 1993; Kim *et al.*, 2003; Halbwirth *et al.*, 2006
G104118		5	YES	AB074486	Honda *et al.*, 2002
M941174		12	NO	CN491664	Henry-Kirk *et al*., 2012
M190489	MdF3'H	6	YES	FJ919632, FJ919633	Han *et al.*, 2010
M286933		14	YES	FJ919631	-
M183682	MdFLS	8	NO	AF119095	Halbwirth *et al.*, 2006
M260404		8	NO	EB137300	Henry-Kirk *et al*., 2012
M229796		14	YES	-	-
M440654	MdDFR	8	NO	AF494392	Venisse *et al.*, 2002
M494976		12	YES	AB074488, AF117268, AF494390, AF494391, AY227728, AY227729	Honda *et al.*, 2002; Venisse *et al.*, 2002; Fischer *et al.*, 2003; Kim *et al.*, 2003
M788934	MdLDOX	6	YES	DQ156905	Fischer *et al.*, 2006
M240641		6	YES	P51091	Davies, 1993
M360447		6	YES	AF117269, DQ381771	Li *et al.*, 2007, Kim *et al.*, 2003
M170162	MdUFGT	8	YES	-	-
M478252*		1	YES	AB074489, AF117267, DQ156906	Honda *et al.*, 2002; Kim *et al.*, 2003; Li *et al.*, 2007
M252292	MdGST	17	YES	-	-
M175055	MdMATE	1	YES	-	-

Identical sequences with different accession numbers are grouped together. Genes without accession numbers and references are identified new in this study.
* This UFGT isoform was identified in our transcriptome profiling study, but not a member of the WGCNA module 'Pink'

Table S6. Promoter analysis using PLACE Signal Scan Search database

Motif Name	Location in the Promoter	Sequence	Function
**MdMYB10 promoter (2585 bp)**			
EECCRCAH1	**-2560**, **-2133**, -1911, -1519, -1378, **-920**, -695, -321	GANTTNC	MYB-binding site.
IBOXCORE	**-1079**, -445	GATAA	Conserved sequence upstream of light-regulated genes.
INRNTPSADB	**-2242**, -1972, -1949, -1835, -1825, -565	YTCANTYY	Light-responsive element.
LTRE1HVBLT49	**-2128**, **-1220**	CCGAAA	Low temperature-responsive element.
LTREATLTI78	**-2156**	ACCGACA	Low temperature-responsive element.
LTRECOREATCOR15	-1661	CCGAC	Core of low temperature-responsive element.
MYB1*AT*	-1537, -1526, **-1180**, **-1127**, **-1077**, **-1015**	WAACCA	MYB-recognition site.
MYB2AT	-861	TAACTG	MYB-binding site.
MYB2CONSENSUSAT	**-2190**, **-1244**, **-1208**, -221	YAACKG	MYB-recognition site.
MYB Core	**-2205**, **-2190**, -2072, -1875, -1664, -1281, -858	CNGTTR	MYB-binding site.
MYB Core *AT*CYCB1	**-2245**, -1671, -1599	AACGG	MYB-binding site.
MYB Plant	-1811, -1275, -790	MACCWAMC	Plant-*MYB* binding site that regulates phenylpropanoid biosynthesis genes.
MYB*ST1*	**-2468**, -1938, **-1186**, -179, -128	GGATA	MYB-binding site.
MYC*AT*RD22	**-2525**, **-1011**	CACATG	MYC-binding site.
MYC Consensus	**-2043**, -1686, **-1123**, **-1106**, **-925**, -469, -221, -101	CANNTG	MYC-recognition site
**MdGST promoter (2724 bp)**			
EECCRCAH1	-1801	GANTTNC	MYB-binding site.
MYB1*AT*	-2309, -1401, -1114, -1102, -541	WAACCA	MYB-recognition site.
MYB2CONSENSUSAT	-2448, -1651, -1167	YAACKG	MYB-recognition site.
MYB Core	-2490, -1946, -1688, -1412, -497	CNGTTR	MYB-binding site.
MYB Core *AT*CYCB1	-1553	AACGG	MYB-binding site.
MYB Plant	-2308	MACCWAMC	Plant-*MYB* binding site that regulates phenylpropanoid biosynthesis genes.
MYBP*ZM*	-518	CCWACC	MYB-binding site of Maize MYB gene specifies red pigmentation of kernel pericarp.
MYB*ST1*	-2379, -1598, -1325	GGATA	MYB-binding site.
MYC*AT*RD22	-2416, -1960	CACATG	MYC-binding site.
MYC Consensus	-2457, -2448, -2425, -2035, -1875, -1651, -1167, -708	CANNTG	MYC-recognition site

Bold single and double underlined locations refer to *cis*-acting elements detected within MR3 and MR7 regions of *MdMYB10* promoter, respectively.

Table S7. Correlations between the MdMYB10 promoter methylation, anthocyanin contents, and gene expression.

	Methylation in MR3 (n=21)		Methylation in MR7 (n=15)	
	r	*P*	*r*	*P*
Anthocyanin content	-0.8002	1.34×10^{-5}	-0.8612	3.73×10^{-5}
MdMYB10-expression	-0.7638	5.59×10^{-5}	-0.8492	6.19×10^{-5}
MdGST-expression	-0.7755	3.63×10^{-5}	-0.7196	2.50×10^{-3}

Table S8. Anthocyanin contents in the skin and flesh tissues of immature and mature fruit of 14 *Malus* accessions

Common Name	Tissue	Immature (S2)							Mature (S4)						
		C-3-Gal	C-3-Glu	C-3-Ara	C-3-Rut	Ukw nonacy	Ukw acy	Total	C-3-Gal	C-3-Glu	C-3-Ara	C-3-Rut	Ukw nonacy	Ukw acy	Total
Vogelcalville	Skin	0	0	0	0	0	0	0	4.0±0.6	0	0	0	0	0	4.0±0.6
Smoothgold		4.8±0.5	0	0	0	0	0	4.8±0.5	5.3±0.7	0	0	0	0	0	5.3±0.7
PRI 1345		0	0	0	0	0	0	0	10.1±0.9	0	0	0	0	0	10.1±0.9
Blondee		4.3±0.4	0	0	0	0	0	4.3±0.4	6.7±0.7	3.7±0.5	3.9±0.4	0	7.5±0.9	7.5±1.1	29.3±4.9
Rose Bud		27.3±3	4.4±0.4	4.8±0.3	0	12.2±2	9.6±1	58±6	31.2±3	5.3±0.5	5.7±0.6	0	14.2±2	13.8±1	70±8
Kidd's D-8		28.4±3	6.5±0.7	8.0±1.1	0	13.4±2	13.6±1	70±8	262±28	8.5±0.9	27.4±3	0	17.8±2	21.1±3	337±37
Galgala		46.7±6	4.2±0.5	7.8±1.1	0	9.5±1.2	9.8±1.2	78±10	432±42	8.5±1.1	44.9±6	0	24.5±3	29.5±4	540±58
Tydeman R.		193±22	6.3±0.8	9.4±1.1	0	19.8±2	16.7±2	245±26	976±102	15.9±2	52.4±6	0	55.1±6	62.3±7	1162±124
Jerseymac		224±25	7.4±0.8	18.1±2	25.8±3	20.8±3	24.3±4	320±39	1389±141	26.1±3	74.2±8	211±25	73.0±9	72.3±10	1845±204
PRI 1236		162±18	5.7±0.7	12.4±2	4.9±0.6	14.2±2	14.4±2	214±29	1638±172	23.8±4	91±11	7.0±0.9	60.7±8	67.4±10	1888±217
Earlibrite D.		1185±98	12.3±1	56.4±5	14.5±1	95.1±7	72.3±9	1435±169	1252±113	14.7±2	61.6±8	17±2	101±12	98±11	1544±186
Red Flesh		372±41	7.0±1	13.9±2	4.2±0.6	48.4±7	31.1±3	477±59	757±86	8.9±1.1	25.3±3	5.8±0.8	75.6±10	48.3±5	921±103
Pink Wood		178±20	4.8±0.8	7.3±1	4.9±0.8	18.3±3	13.6±2	227±28	833±108	8.3±1	18.7±3	6.5±1	49.2±6	31.3±4	947±118
Eleyi		756±86	9.7±1.3	18.2±3	5.2±0.8	95.4±13	64.9±9	950±118	929±105	13.4±2	20.4±3	5.6±0.9	112±15	70.3±10	1151±132
Vogelcalville	Flesh	0	0	0	0	0	0	0	0	0	0	0	0	0	0
Smoothgold		0	0	0	0	0	0	0	0	0	0	0	0	0	0
PRI 1345		0	0	0	0	0	0	0	0	0	0	0	0	0	0
Blondee		0	0	0	0	0	0	0	0	0	0	0	0	0	0
Rose Bud		53.7±5	4.9±0.5	6.0±0.5	0	36.1±4	26.3±3	127±13	88.2±9	6.9±0.7	8.0±0.8	0	43.9±4	38.2±4	185±19
Kidd's D-8		0	0	0	0	0	0	0	0	0	0	0	0	0	0
Galgala		0	0	0	0	0	0	0	4.1±0.6	0	0	0	0	0	4.1±0.6
Tydeman R.		0	0	0	0	0	0	0	4.1±0.7	0	0	0	0	0	4.1±0.7
Jerseymac		0	0	0	0	0	0	0	6.5±0.8	0	0	0	0	0	5.7±0.9
PRI 1236		0	0	0	0	0	0	0	6.6±0.9	0	0	0	0	0	6.6±0.9
Earlibrite D.		0	0	0	0	0	0	0	5.7±0.7	0	0	0	0	0	5.7±0.7
Red Flesh		66.7±9.1	4.4±0.7	5.1±0.8	5.8±0.7	7.8±0.9	21.3±2	111±15	84.3±10	5.5±0.9	6.0±0.7	9.7±1.1	11.5±2	31.2±3	148±18
Pink Wood		91.5±13	3.8±0.6	4.4±0.7	3.5±0.5	13.2±2	7.3±1.1	124±15	125±18	4.1±0.7	5.7±0.9	4.7±0.8	16.1±3	11.1±2	166±20
Eleyi		111±15	4.9±0.8	4.9±0.8	4.5±0.7	9.9±1.3	9.8±1.2	145±19	216±26	7.3±1.2	8.8±1.1	5.0±0.8	62.8±8	41.7±6	342±42

Anthocyanin contents (μg g^{-1} DW). C-3-Gal, C-3-Glu, C-3-Ara, Ukw nonacy, and Ukw acyl refer to cyanidin 3-galactoside, cyanidin 3-glucoside, cyanidin 3-arabinoside, unknown nonacylated, and unknown acylated anthocyanins, respectively.

Table S9. Pearson's correlation coefficient between the expression profile of ten selected genes and the contents of total anthocyanin and individual anthocyanins.

Tissue type	Anthocyanin	MdCHI	MdCHS	MdDFR	MdF3'H	MdF3H	MdGST	MdLDOX	MdMYB10	MdUFGT	MdWD40
Immature fruit skin	Total-Anthocyanin	-0.003^{NS}	0.799**	0.708**	0.709**	0.765**	0.835**	0.390^{NS}	0.745**	0.785**	0.667**
	Cy-3-Galactoside	-0.019^{NS}	0.784**	0.692**	0.693**	0.752**	0.820**	0.376^{NS}	0.726**	0.770**	0.649*
	Cy-3-Glucoside	0.242^{NS}	0.878**	0.834**	0.806**	0.809**	0.971**	0.590*	0.922**	0.935**	0.856**
	Cy-3-Arabinoside	-0.013^{NS}	0.677**	0.594*	0.550*	0.620**	0.814**	0.456^{NS}	0.667**	0.775**	0.606*
	Cy-3-Rutinoside	-0.138^{NS}	0.477^{NS}	0.427^{NS}	0.402^{NS}	0.416^{NS}	0.545*	0.039^{NS}	0.436^{NS}	0.530^{NS}	0.489^{NS}
Mature fruit skin	Total-Anthocyanin	0.049^{NS}	0.873**	0.821**	0.825**	0.796**	0.955**	0.104^{NS}	0.862**	0.917**	0.867**
	Cy-3-Galactoside	0.053^{NS}	0.876**	0.829**	0.828**	0.807**	0.956**	0.100^{NS}	0.865**	0.919**	0.876**
	Cy-3-Glucoside	0.068^{NS}	0.800**	0.739**	0.742**	0.686**	0.923**	0.145^{NS}	0.804**	0.850**	0.768**
	Cy-3-Arabinoside	0.106^{NS}	0.698**	0.638**	0.621*	0.553*	0.873**	0.224^{NS}	0.707**	0.777**	0.692**
	Cy-3-Rutinoside	-0.140^{NS}	0.294^{NS}	0.226^{NS}	0.268^{NS}	0.180^{NS}	0.372^{NS}	-0.141^{NS}	0.301^{NS}	0.312^{NS}	0.225^{NS}
Immature fruit flesh	Total-Anthocyanin	0.820**	0.944**	0.911**	0.939**	0.937**	0.928**	0.849**	0.953**	0.875**	0.972**
	Cy-3-Galactoside	0.917**	0.920**	0.916**	0.901**	0.912**	0.908**	0.839**	0.911**	0.880**	0.995**
	Cy-3-Glucoside	0.756**	0.939**	0.892**	0.937**	0.931**	0.921**	0.830**	0.952**	0.863**	0.950**
	Cy-3-Arabinoside	0.714**	0.927**	0.873**	0.931**	0.921**	0.911**	0.813**	0.949**	0.846**	0.921**
	Cy-3-Rutinoside	0.737**	0.769**	0.755**	0.723**	0.753**	0.768**	0.601*	0.761**	0.806**	0.862**
Mature fruit flesh	Total-Anthocyanin	0.824**	0.936**	0.902**	0.893**	0.893**	0.859**	0.916**	0.927**	0.928**	0.969**
	Cy-3-Galactoside	0.881**	0.935**	0.900**	0.875**	0.877**	0.860**	0.935**	0.912**	0.951**	0.984**
	Cy-3-Glucoside	0.693**	0.899**	0.906**	0.929**	0.926**	0.849**	0.881**	0.953**	0.865**	0.917**
	Cy-3-Arabinoside	0.731**	0.908**	0.916**	0.935**	0.934**	0.858**	0.898**	0.960**	0.884**	0.934**
	Cy-3-Rutinoside	0.578*	0.701**	0.745**	0.698**	0.710**	0.689**	0.795**	0.738**	0.740**	0.771**

Statistically significant differences represented by probability levels (P<0.05) and (P<0.01) are indicated by (*) and (**), respectively. NS, non-significant (P>0.05). [n=14, r0.05= 0.532, r0.01= 0.661].

Supplemental Figure Legends

Fig. S1. Overview of the trees and fruit of 'Kidd's D-8' and 'Blondee'. (A) View of 5-year old KID and BLO trees showing red and yellow fruit production, respectively. (B) Close-up view of red-color KID fruit and yellow-color BLO fruit.

Fig. S2. HPLC chromatogram traces of sample Jerseymac's mature skin. Peaks were annotated with retention time and individual anthocyanin name. mAU: milli absorption units.

Fig. S3. Evaluation of the KID and BLO parent-mutant relationships. (A) Polyacrylamide gel electrophoresis images of DNA fingerprinting by SSR markers. The images show 12 of the 16 markers analyzed, including 11-informative and 1-non-informative (C4251). The other four markers were also non-informative (not shown). Note that each cultivar was represented by three lanes with 'Fuji' in the middle. (B) Fruit weight of KID and BLO at four stages. Asterisk stars stand for significant difference (P<0.05). (C) Cornell Starch Index of KID and BLO at harvest.

Fig. S4. Venn diagram representation of the number of all expressed genes identified by RNA-seq analysis in the skin tissues of BLO and KID fruit at varying developmental stages. The cutoff is RPKM>1.0. (A) and (B) Number of all expressed genes at S1 through S4 in BLO and KID, respectively. (C) and (D) Number of all expressed genes in BLO and KID at S1 and S2 and at S3 and S4, respectively.

Fig. S5. Screening of *MdGST* promoter regions for different methylation levels between KID and BLO using McrBC-PCR. The *g*DNA from the skin of mature fruit (S4) collected in 2013 and 2014 was used. The promoter region was divided into five fragments (named GR1 through GR5), and the location of each fragment is indicated. The number of cycles necessary for exponential, but non-saturated, PCR amplification was determined for each region using the negative control template (GTP replaced by water). Letter M and C indicate the McrBC digestion reactions with or without GTP (i.e. negative control), respectively. Reactions were performed in three independent triplicate PCR reactions driven from three independent digestions.

Fig. S6. Hierarchical cluster analysis (Ward's method) of fruit scan and flesh samples from 14 *Malus* accessions. The analysis was conducted using the relative gene expression (qRT-PCR) data of ten genes from the WGCNA module 'Pink'. The numbers show the largest distance between samples in an individual group. (A) Immature fruit skin; (B) Mature fruit skin; (C) Immature fruit flesh; (D) Mature fruit flesh.

Fig. S7. Kruskal-Wallis QTL mapping of the yellow-scan fruit trait in 'Royal Gala' (A) and PI613988 (B). The horizontal axes show the genetic maps of Linkage Group 9 as previously reported (Wang *et al*., 2012). Marker CN444542 is linked to the fruit color trait with the most significant K-W statistics (P<0.00001), i.e. 40.8 and 55.7 in the 'Royal Gala' in PI613988 genomes, respectively. cM: centiMorgan.

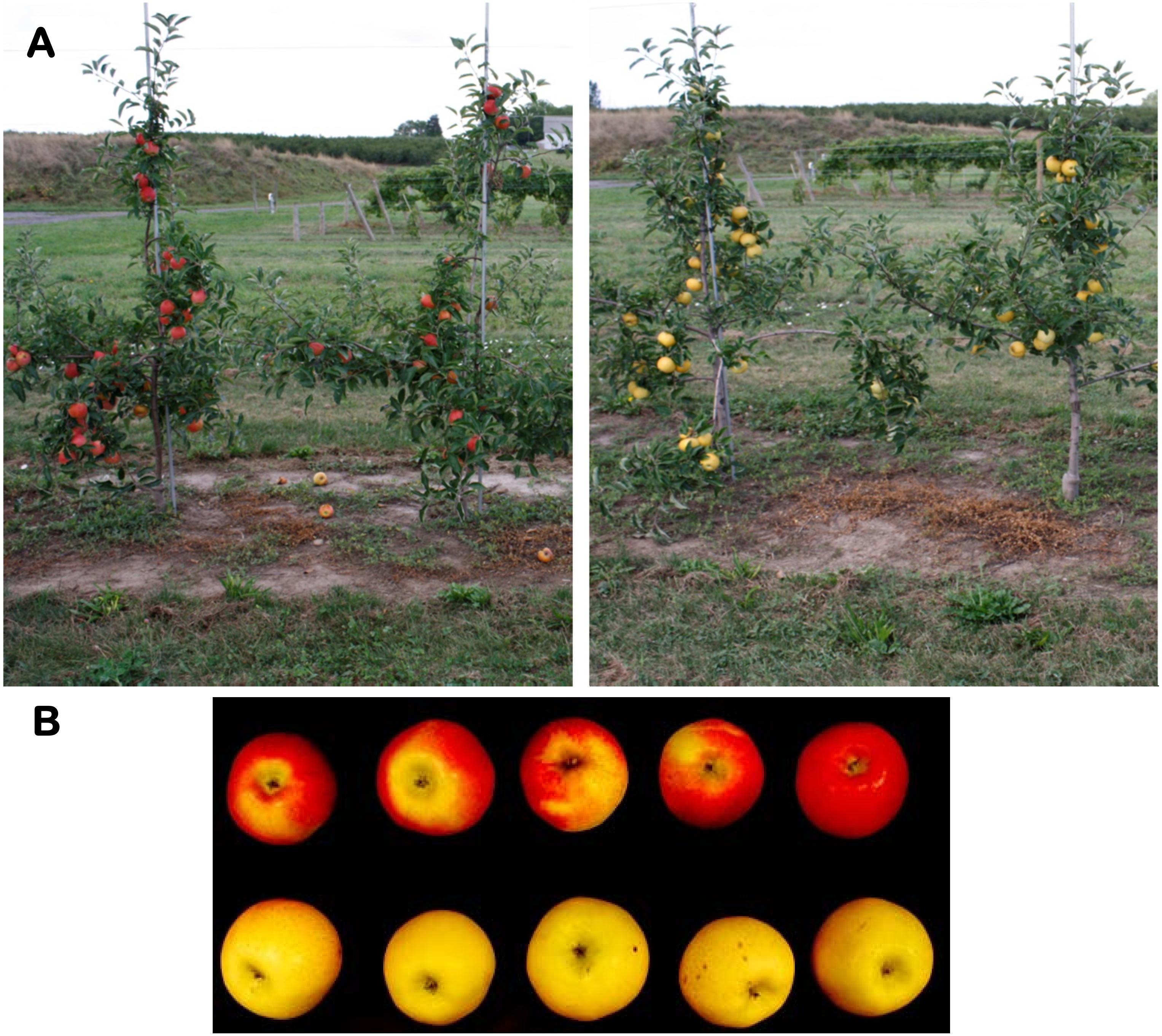

El-Sharkawy, Liang and Xu: Fig.S1

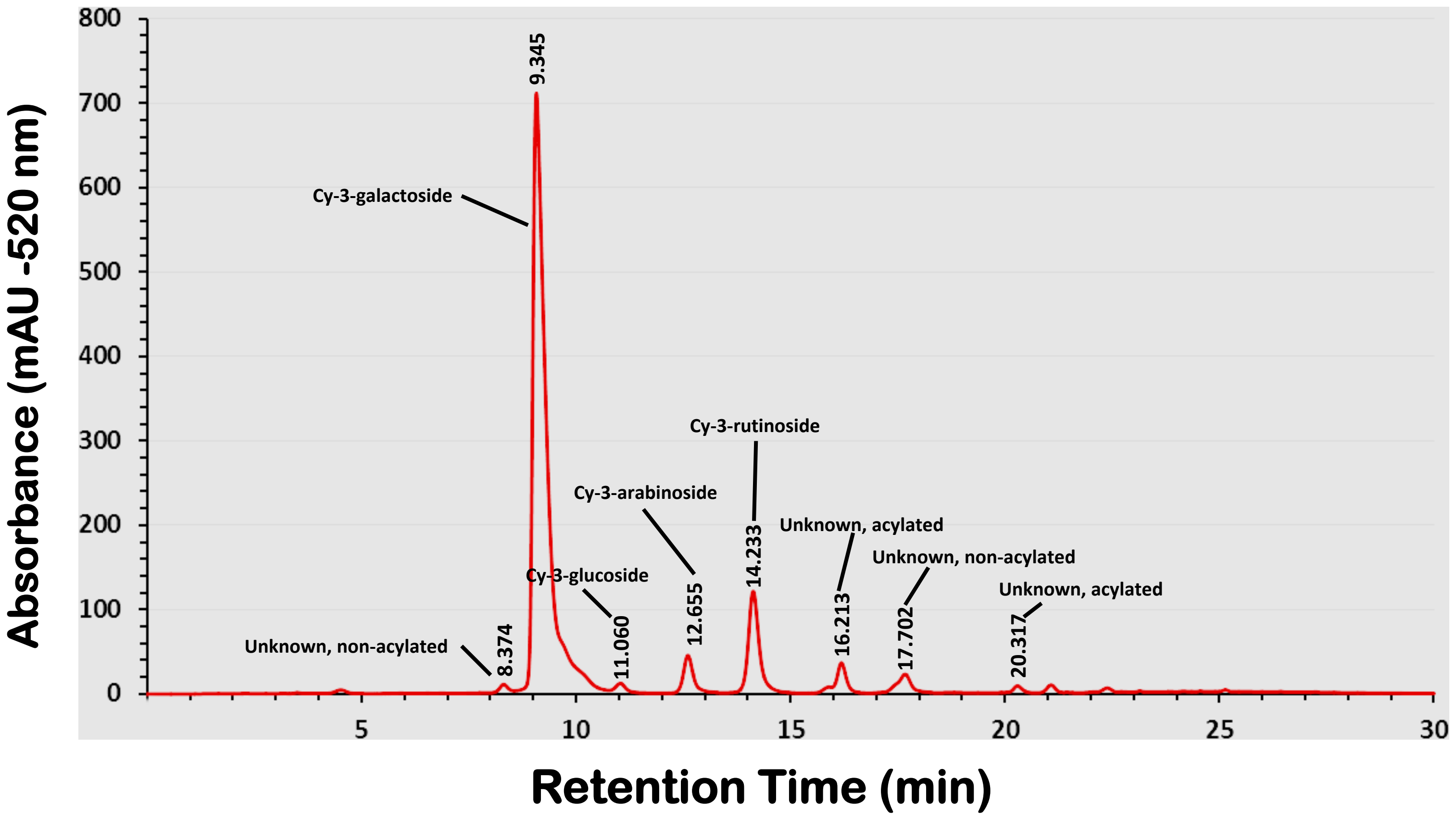

El-Sharkawy, Liang and Xu: Fig.S2

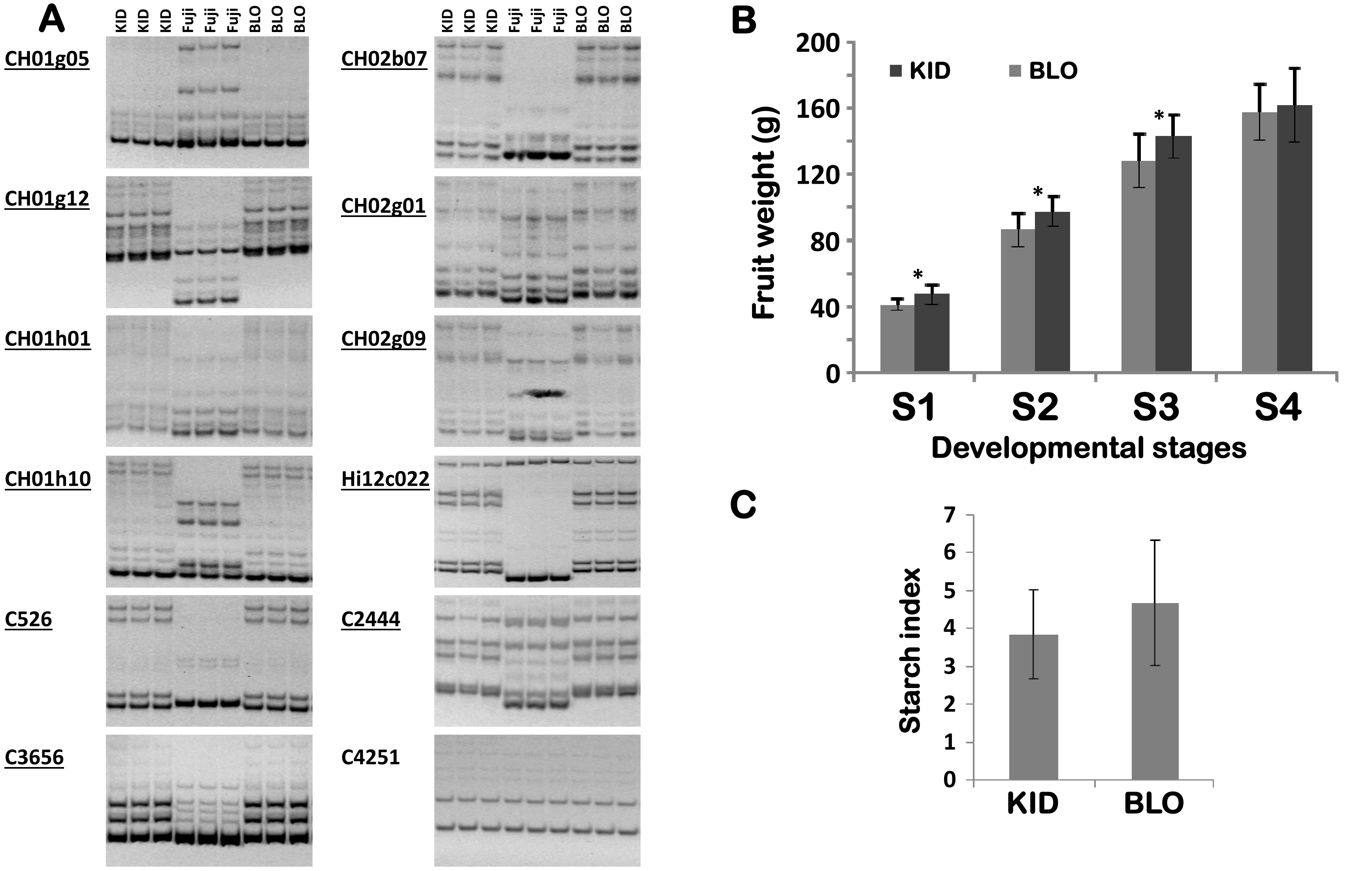

El-Sharkawy, Liang and Xu: Fig.S3

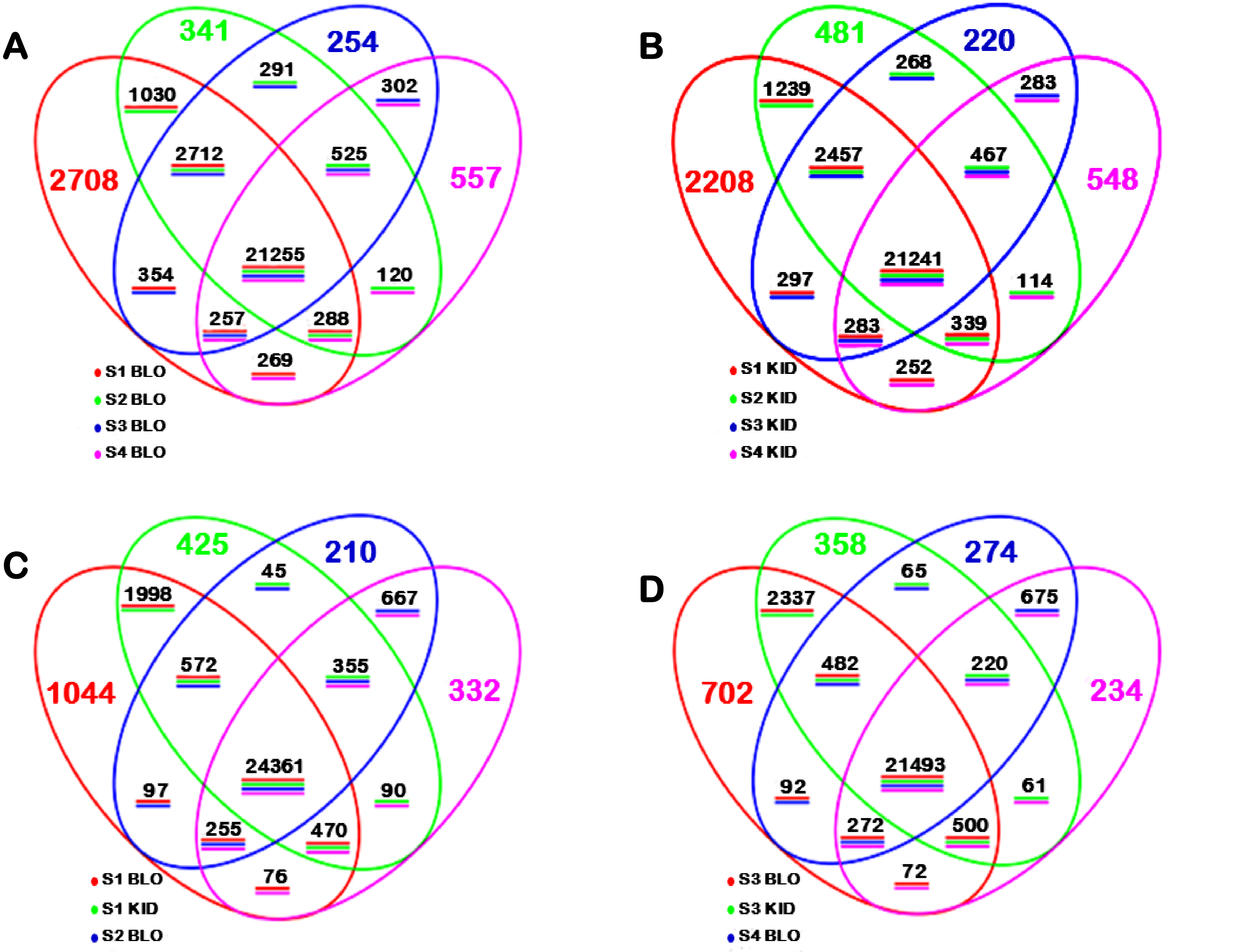

El-Sharkawy, Liang and Xu: Fig.S4

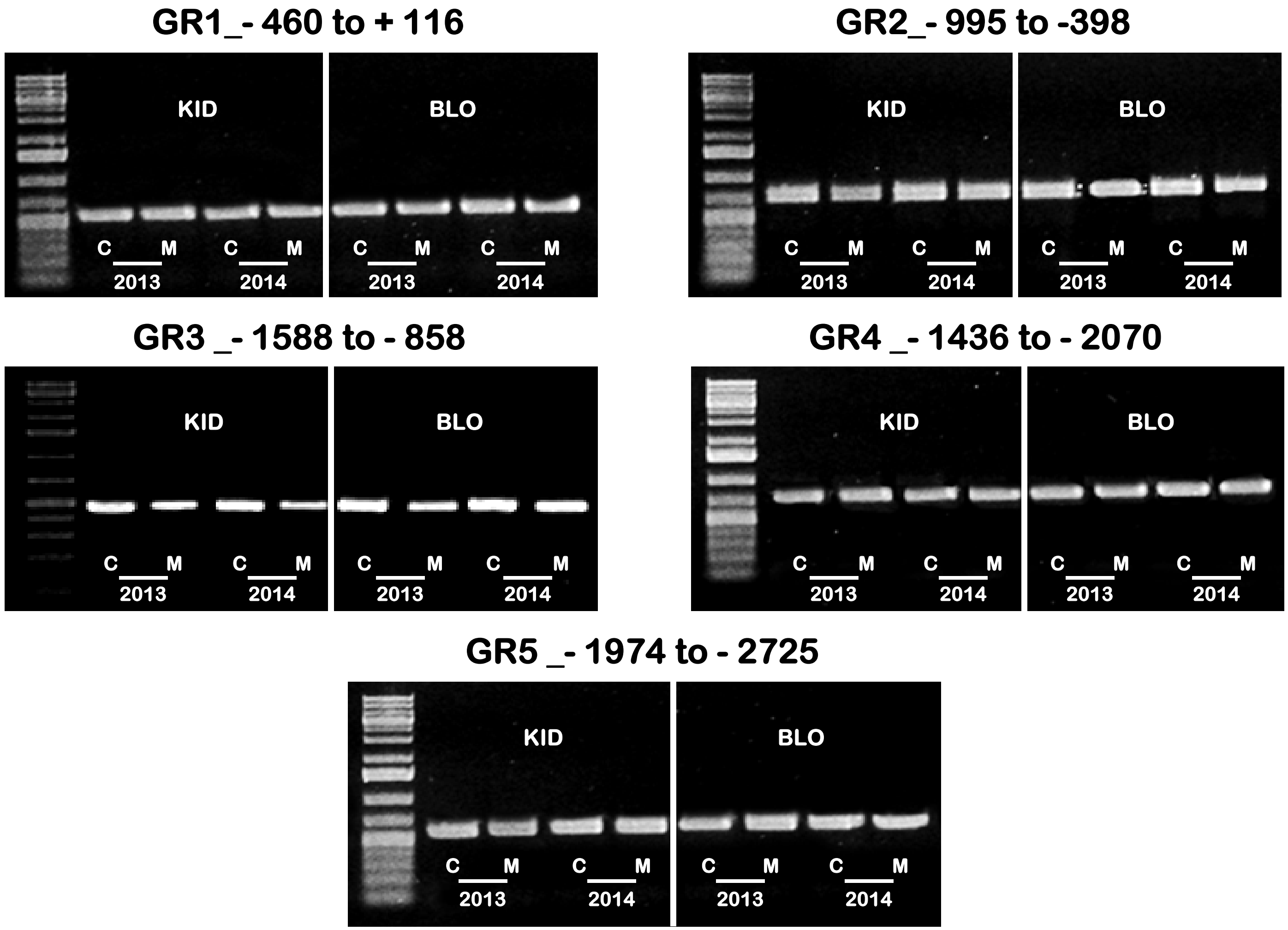

El-Sharkawy, Liang and Xu: Fig.S5

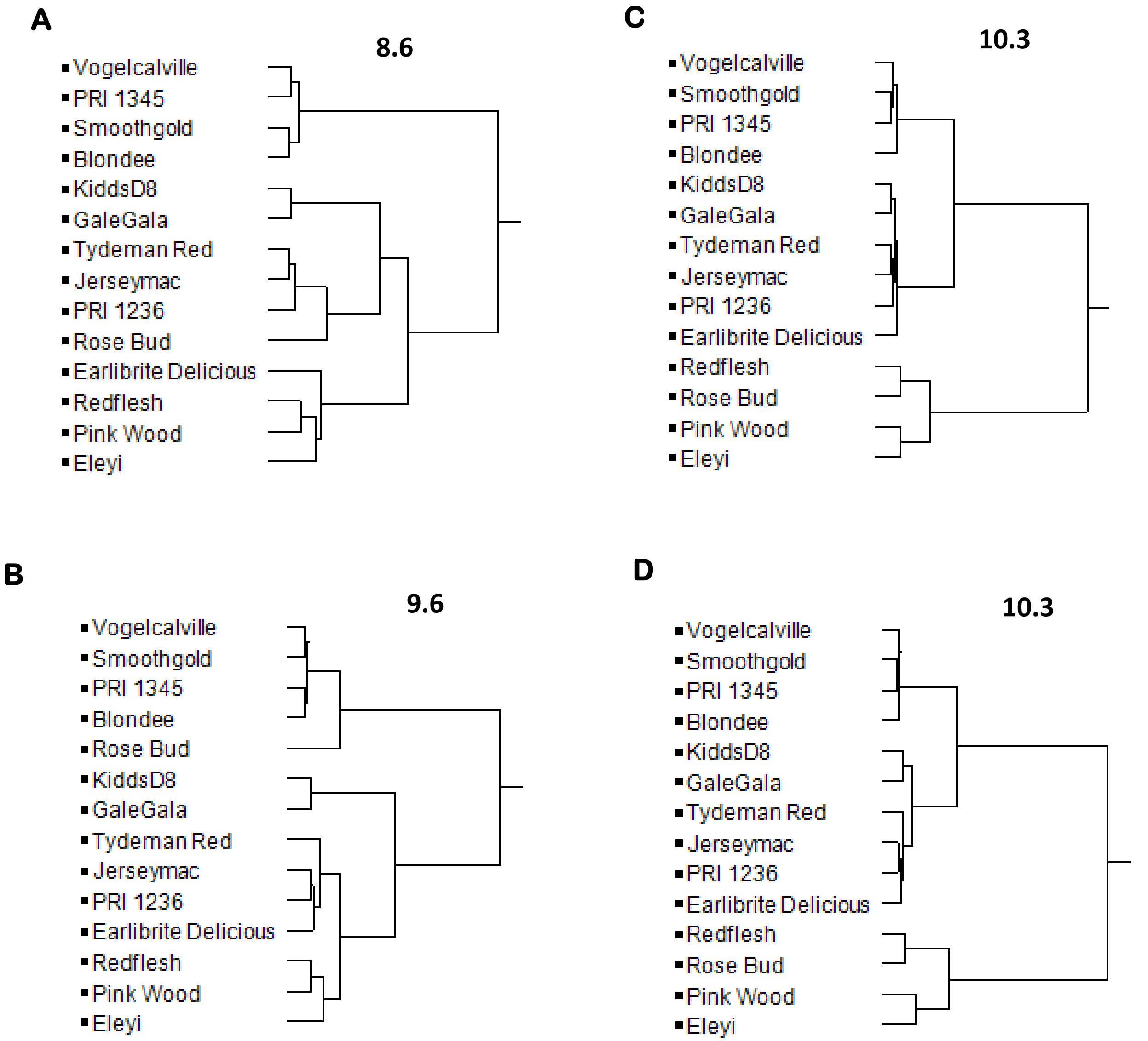

El-Sharkawy, Liang and Xu: Fig.S6

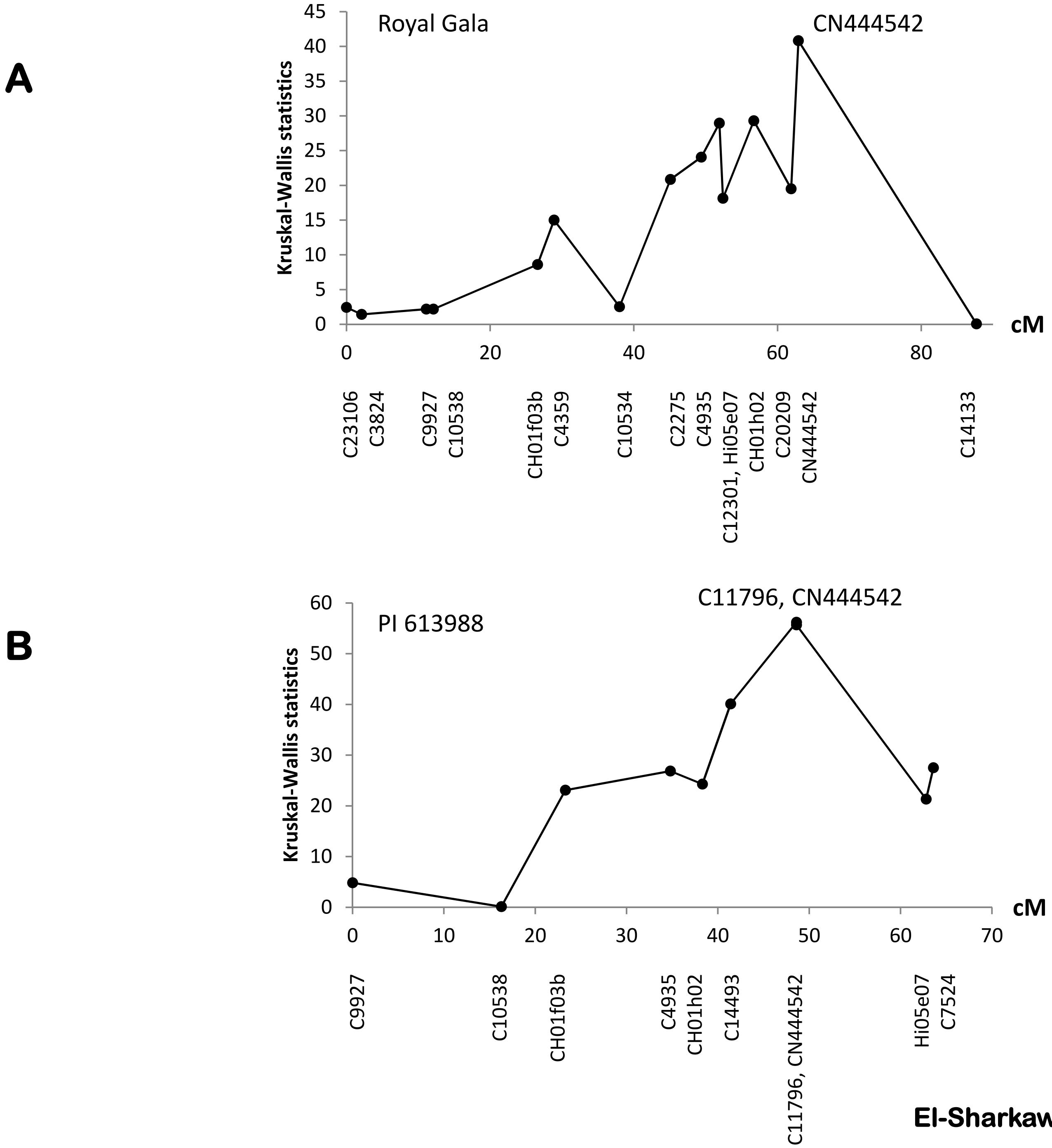

El-Sharkawy, Liang and Xu: Fig.S7
